# GMP-Compliant Manufacturing of TRUCKs: CAR T Cells targeting GD_2_ and Releasing Inducible IL-18

**DOI:** 10.3389/fimmu.2022.839783

**Published:** 2022-03-24

**Authors:** Wolfgang Glienke, Anna Christina Dragon, Katharina Zimmermann, Alexandra Martyniszyn-Eiben, Mira Mertens, Hinrich Abken, Claudia Rossig, Bianca Altvater, Krasimira Aleksandrova, Lubomir Arseniev, Christina Kloth, Andriana Stamopoulou, Thomas Moritz, Holger N. Lode, Nikolai Siebert, Rainer Blasczyk, Lilia Goudeva, Axel Schambach, Ulrike Köhl, Britta Eiz-Vesper, Ruth Esser

**Affiliations:** ^1^ ATMP-GMP Development Unit, Institute of Cellular Therapeutics, Integrated Research and Treatment Center for Transplantation, Hannover Medical School, Hannover, Germany; ^2^ Institute of Transfusion Medicine and Transplant Engineering, Hannover Medical School, Hannover, Germany; ^3^ Division of Hematology/Oncology, Institute of Experimental Hematology, Hannover Medical School, Hannover, Germany; ^4^ Leibniz Institute for Immunotherapy, Div Genetic Immunotherapy, Regensburg, Germany; ^5^ Department of Pediatric Hematology and Oncology, University Children’s Hospital Muenster, Muenster, Germany; ^6^ Cellular Therapy Center, Institute of Cellular Therapeutics, Hannover Medical School, Hannover, Germany; ^7^ Department of Pediatric Hematology and Oncology, University Medicine Greifswald, Greifswald, Germany; ^8^ Boston Children’s Hospital, Harvard Medical School, Boston, MA, United States; ^9^ Fraunhofer Institute for Cell Therapy and Immunology (IZI), Leipzig, Germany; ^10^ Clinical Immunology, University of Leipzig, Leipzig, Germany

**Keywords:** TRUCK, IL-18, GD2-CAR, Prodigy, GMP, 4^th^ generation CAR

## Abstract

Chimeric antigen receptor (CAR)-engineered T cells can be highly effective in the treatment of hematological malignancies, but mostly fail in the treatment of solid tumors. Thus, approaches using 4^th^ advanced CAR T cells secreting immunomodulatory cytokines upon CAR signaling, known as TRUCKs (“*T cells redirected for universal cytokine-mediated killing*”), are currently under investigation. Based on our previous development and validation of automated and closed processing for GMP-compliant manufacturing of CAR T cells, we here present the proof of feasibility for translation of this method to TRUCKs. We generated IL-18-secreting TRUCKs targeting the tumor antigen GD_2_ using the CliniMACS Prodigy^®^ system using a recently described “all-in-one” lentiviral vector combining constitutive anti-GD_2_ CAR expression and inducible IL-18. Starting with 0.84 x 10^8^ and 0.91 x 10^8^ T cells after enrichment of CD4^+^ and CD8^+^ we reached 68.3-fold and 71.4-fold T cell expansion rates, respectively, in two independent runs. Transduction efficiencies of 77.7% and 55.1% was obtained, and yields of 4.5 x 10^9^ and 3.6 x 10^9^ engineered T cells from the two donors, respectively, within 12 days. Preclinical characterization demonstrated antigen-specific GD_2_-CAR mediated activation after co-cultivation with GD_2_-expressing target cells. The functional capacities of the clinical-scale manufactured TRUCKs were similar to TRUCKs generated in laboratory-scale and were not impeded by cryopreservation. IL-18 TRUCKs were activated in an antigen-specific manner by co-cultivation with GD_2_-expressing target cells indicated by an increased expression of activation markers (e.g. CD25, CD69) on both CD4^+^ and CD8^+^ T cells and an enhanced release of pro-inflammatory cytokines and cytolytic mediators (e.g. IL-2, granzyme B, IFN-γ, perforin, TNF-α). Manufactured TRUCKs showed a specific cytotoxicity towards GD_2_-expressing target cells indicated by lactate dehydrogenase (LDH) release, a decrease of target cell numbers, microscopic detection of cytotoxic clusters and detachment of target cells in real-time impedance measurements (xCELLigence). Following antigen-specific CAR activation of TRUCKs, CAR-triggered release IL-18 was induced, and the cytokine was biologically active, as demonstrated in migration assays revealing specific attraction of monocytes and NK cells by supernatants of TRUCKs co-cultured with GD_2_-expressing target cells. In conclusion, GMP-compliant manufacturing of TRUCKs is feasible and delivers high quality T cell products.

## Introduction

One of the most significant recent developments in cancer therapy is the CAR T cell technology. To enable and improve CAR T cell proliferation, anti-tumor activity, and *in vivo* persistence, advanced generations of CARs have been developed (1). A promising strategy to target solid tumors with their phenotypic heterogeneity has led to the fourth generation of CARs also known as TRUCKs or armored CARs (2, 3). TRUCKs are CAR T cells that release a transgenic protein upon CAR engagement of cognate antigen and signaling. TRUCKs are thereby used as “living factories” to produce and deposit substances with anti-tumor activity in the targeted tissue. These factors include cytokines, such as IL (interleukin)-12 and IL-18, but also enzymes and costimulatory ligands augmenting T cell activation. Innate immune cells are attracted and activated by IL-12 or IL-18 (4) to eliminate antigen-low expressing or antigen-negative cancer cells within the tumor (2). CAR T cells engineered with inducible IL-18 release improve T cell effector functions towards superior activity against pancreatic and lung tumors in mice that were refractory to CAR T cells without cytokines (5, 6). In this study we focused on the manufacturing of TRUCKs targeting disialoganglioside GD_2_. Physiological expression of GD_2_ is restricted to low densities on neurons, skin melanocytes and peripheral pain fibers (7). GD_2_ is highly and consistently expressed in childhood cancer neuroblastoma and can be found on the cell surface of other solid cancer entities including breast cancer (8), osteosarcoma (9), melanoma (10), glioblastoma (11), small cell lung cancer (12), retinoblastoma (13), soft tissue sarcoma (14) and Ewing sarcoma (15, 16).

GD_2_ therefore is a promising target for redirected immunotherapy. Initial GD_2_-CAR T cell clinical studies targeting neuroblastoma by first to third generation CAR T cells showed moderate or transient anti-tumor responses but failed to produce sustained remissions, emphasizing the need to modulate the T cell response (17–19). With the increasing expectation of GD_2_ as a broad target for CAR T cell therapy and the expected benefit in applying TRUCKs with transgenic IL-18 release, there is a need to manufacture such cellular medicinal products in a safe, validated and reproducible fashion. CAR T cell manufacturing for clinical use is a complex process and places high standard demands on safety, quality and efficacy. Chemistry, Manufacturing and Controls (CMC) even in the preclinical phase of drug development includes significant quality attributes and critical process parameters, including cell composition and transduction efficiency, assessment of potency, product sterility, process validation, stability and production at multiple manufacturing sites (20). The CliniMACS Prodigy^®^ (Miltenyi Biotec B.V. & Co. KG) allows *ex vivo* magnetic bead-based cell separation followed by activation, transduction, expansion, final formulation and sampling of T cells in one device leading to robust, reproducible and automated, supervised cost-effective manufacturing processes [Process of CAR T cell Therapy in Europe EHA Guidance Document, 2019 (21)]. The feasibility of the T cell transduction (TCT) process for use in automated and closed GMP-compliant manufacturing of CAR T cells on the CliniMACS Prodigy^®^ platform, as shown by us and others (22–25) is here extended to the manufacturing of IL-18 TRUCKs targeting GD_2_ as an example. Preclinical characterization showed equivalent quality and function of the final clinical-scale products compared to manually produced IL-18 TRUCKs in laboratory-scale.

## Materials and Methods

### Human Sample Materials

For the manufacturing of TRUCKs in a clinical-scale process (n=2) on the CliniMACS Prodigy^®^ (Miltenyi Biotec, Bergisch Gladbach, Germany) and for the manufacturing of laboratory-scale TRUCKs (n=3) lymphapheresis products from two healthy donors (D1 and D2) were obtained from the Institute for Transfusion Medicine of Hannover Medical School (MHH) after donors’ written informed consent. According to standard donation requirements, the donors had no signs of acute infection and no previous history of blood transfusion.

### IL-18 TRUCK Construct and Production of Lentiviral Supernatants

The generation of the lentiviral IL-18 TRUCK SIN vector was described previously (26). In brief, the lentiviral 3^rd^ generation SIN vector pCCL.PPT.NFATsyn.hIL18.PGK.GD2CAR.PRE was used. The human codon-usage optimized ORF second-generation CAR containing scFv (14.G2a), human IgG1- hinge, CD28 transmembrane, CD137 (4-1BB), and CD3ζ signaling domains was flanked by restriction enzymes AgeI and SalI and cloned into the lentiviral “all-in-one” SIN vector driven by an hPGK promoter (27). A schematic map of the construct is presented in **Supplement Figure S8**. The construct was confirmed by sequencing (Microsynth SeqLab, Germany). Lentiviral vector particles were generated as described previously (26, 28). Briefly, 5 × 10^6^ 293T cells were used for calcium phosphate transfection in the presence of 25 µM chloroquine. For transfection, the following plasmids were used: lentiviral vector plasmid (10 µg), pcDNA3.HIV-1.GP.4 × CTE (lentiviral gag/pol) (12 µg) (29), pRSV-Rev (5 µg; kindly provided by T. Hope, Northwestern University Chicago, IL), and VSVg-encoding pMD.G (1.5 µg) (30). For better standardization, pcDNA3.HIV-1.GP.4×CTE, pRSV-Rev and pMD.G were produced and purified by PlasmidFactory (Bielefeld, Germany).After 36 h and 48 h of transfection, supernatants were harvested and concentrated *via* ultracentrifugation at 4^°^C and 13,238× g or 82,740× g for 16 h or 2 h, respectively. The particles were resuspended in TexMACS™ GMP medium. Lentiviral supernatant was titrated in HT1080 fibroblasts *via* spinoculation-mediated transduction, i. e. 1 × 10^5^ cells were seeded, the supernatant containing viral particles and 4 µg/ml protamine sulfate (Sigma-Aldrich, St. Louis, USA) were added and cells were centrifuged (1 h, 800× g, 37^°^C). Three days post transduction, transduction efficiency was determined *via* flow cytometric staining of GD_2_-CAR expression and functional viral vector titers were calculated from samples with GD_2_-CAR expression percentages of ≤ 30% to avoid cells with multiple integrations (26, 31).

### GMP-Compliant Manufacturing of IL-18 TRUCKs Targeting GD_2_ With CliniMACS Prodigy^®^ (Clinical-Scale Process)

**Figure 1:**
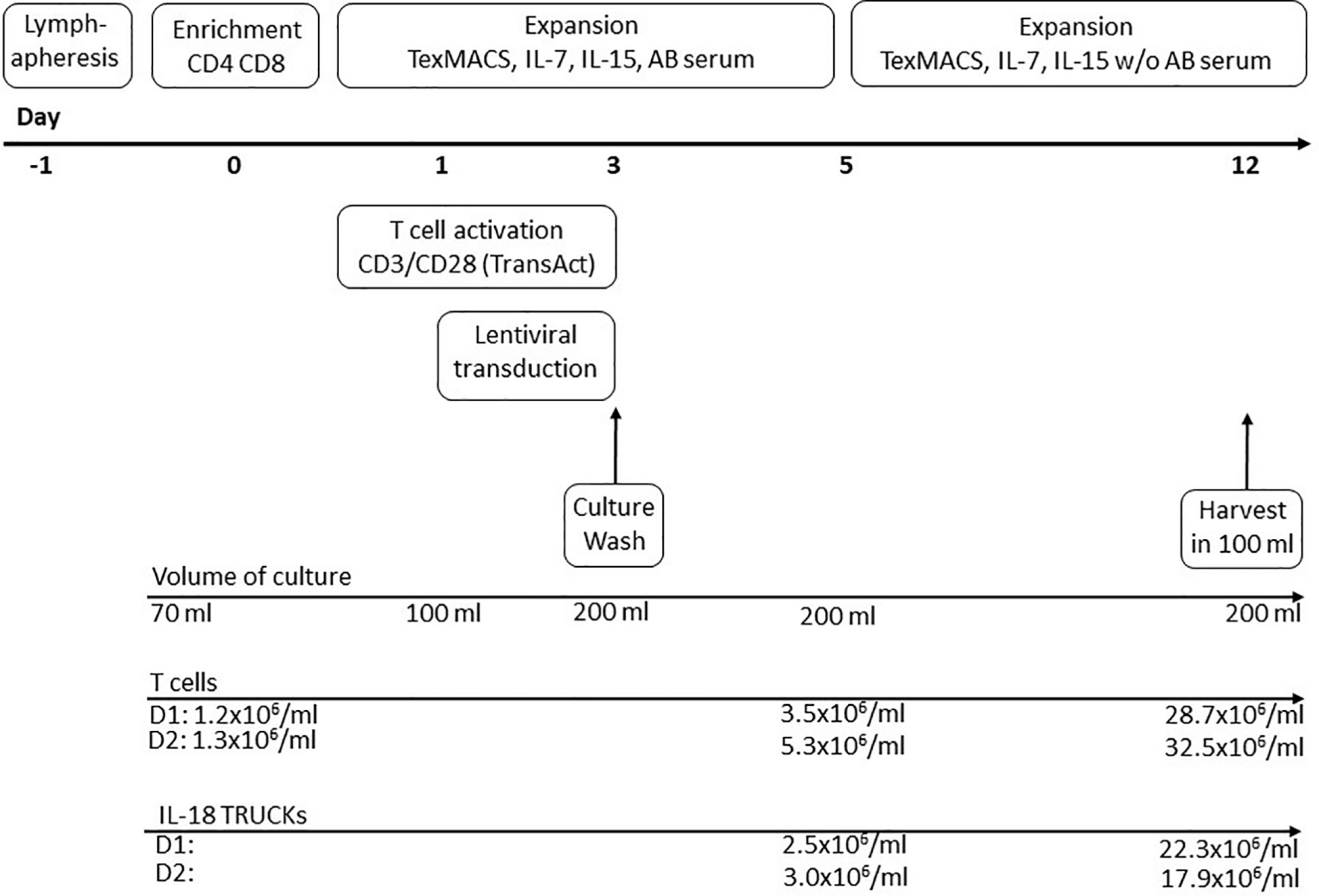
Schematic diagram of producing IL-18 TRUCKs targeting GD_2_ utilizing the CliniMACS Prodigy^®^ automated cell processor (clinical-scale process). The entire 12-day process from lymphapheresis material to formulated final products was performed using the CliniMACS Prodigy^®^. **Day -1**: Overnight storage of the lymphapheresis at 4°C. **Day 0**: Immunomagnetic enrichment of CD4^+^ and CD8^+^ cells. Start of T cell activation with CD3/CD28 TransAct Beads (day 0-3) and cultivation in TexMACS™ basal medium supplemented with IL-7, IL-15 and human AB serum until day 5. **Day 1-3**: Lentiviral transduction. **Day 3**: Culture wash and start of culture agitation. **Day 5**: Switch to TexMACS™ basal medium supplemented with IL-7, IL-15 without human AB serum for following media exchanges. **Day 12**: Wash of cells and harvest in 100 ml formulation buffer for following cryopreservation. During T cell expansion several feeding steps by medium exchange were included in the activity matrix.

GMP-compliant manufacturing of IL-18 TRUCKs was performed using the CliniMACS Prodigy^®^ platform, which allows for automated cell processing in a closed system controlled by operating software version V1.3 and process software for T cell transduction (TCT) version V2.0 (released). For overview of clinical-scale process see **Figure 1**. Within the scope of the automatically running process, the input of different variable process parameters like time points of transduction, media exchange, culture wash, harvesting and volume of media exchange is possible. Buffer, media, starting cell material and vector were connected directly to TS520 *via* sterile tubing welder device (TSCDII Terumo BCT).

The applied materials were either medicinal products with a marketing approval (HSA, PEI.H.03272.01.1), GMP-grade reagents and tubing sets from Miltenyi Biotec (designed following the recommendations of USP <1043> on ancillary materials and/or compliant with the requirements laid down in the Ph. Eur. Chapter 5.2.12, where applicable), or approved medical devices (DMSO, Composol, SSP+, transfer bags, connections, syringes). The pool-Human Serum P-HS/Tü was purchased from the Centre for Clinical Transfusion Medicine Tübingen/ZKT and certified as suitable for manufacturing of pharmaceutical products. The single non-regulated reagent was the vector, designed and produced at the Institute of Experimental Hematology, Hannover Medical School, Division of Hematology/Oncology. Detailed information regarding the materials was recorded, including the supplier, lot number, and expiration date. Starting material for manufacturing of IL-18 TRUCKs were CD3+ T cells derived from a non-mobilized lymphapheresis. Cell processing started within 24 h after product collection with immunomagnetic enrichment of 1 x 10^9^ CD4+ and CD8+ T cells using CliniMACS^®^ CD4 Reagent, CliniMACS^®^ CD8 Reagent and CliniMACS^®^ PBS/EDTA buffer supplemented with human serum albumin (HSA, Human albumin 200 g/l Baxalta, Shire Deutschland GmbH, Berlin, Germany) to a final concentration of 0.5%. For cultivation, the basal TexMACS™ GMP medium was supplemented with 12.5 ng/ml MACS GMP Recombinant Human IL-7, 12.5 ng/ml MACS GMP Recombinant Human IL-15 and 3% heat-inactivated human AB serum (pool-human serum P-HS/Tü, Centre for Clinical Transfusion Medicine Tübingen/ZKT, Germany) until day 5. T cells were activated for 72 h (day 0 to day 3) with CD3/CD28 MACS GMP T Cell TransAct Beads. On day 1 of culture transduction took place by adding 10 ml lentiviral vector with a multiplicity of infection (MOI) of 29 D1 and 10 D2 in total volume of 100 ml. At day 3, CD3/CD28 T cell TransAct beads and non-bound vector were washed out (culture wash) and culture volume increased to 200 ml. The culture was fed every 12 – 24 h after day 5 of culture. Hereby the concentration of AB serum in the culture was reduced continuously using supplemented TexMACS™ GMP medium without AB serum for further medium exchange. On day of harvest (day 12), cells were formulated in Composol PS (Fresenius Kabi Deutschland GmbH, Bad Homburg, Germany) with 2.86% (w/v) HSA (D1) or SSP+ (D2) (Maco Pharma International GmbH, Langen, Germany) with 3.33% HSA for later cryopreservation. During cultivation, the temperature and atmosphere was maintained at 37°C with 5% CO2. After 3 days of static culture shaking modus was activated (culture agitation) enabling high cell concentrations in the limited volume of the CentriCult Chamber.

### Monitoring of Culture and In-Process Controls

Total cell number and viability was analyzed by flow cytometric analysis as described below. The glucose concentration of the cell-free culture supernatant was determined with the blood glucose meter Accu-Chek^®^ Aviva (Roche, Mannheim, Germany). For analysis of pH during cell cultivation pH-indicator strips MColorpHast™ pH 6.5 - 10.0 (Merck Millipore, Darmstadt, Germany) were used.

### Flow Cytometric Characterization of IL-18 TRUCKs Manufactured in Clinical-Scale Process

Flow cytometric analysis was performed by using anti-human monoclonal antibodies. Cellular composition antibody table is shown in **Supplementary Table 1**. Transduction efficiency was determined by GD_2_-CAR detection with the antibody Ganglidiomab, which was conjugated with Phycoerythrin (PE) by Miltenyi Biotec, herein after referred to as Ganglidiomab-PE. The antigen-specific single-chain variable fragment (scFv) of the GD_2_-CAR is derived from the anti-GD_2_ antibody 14G2a (32, 33). Ganglidiomab is a monoclonal anti-idiotype antibody to 14G2a and therefore allows for a direct detection of the GD_2_-CAR on cells. Subsequently antibody staining (10 min at RT) cells were incubated (10 min at RT) with freshly prepared red blood cell lysis solution (Miltenyi Biotec). T cell phenotype antibody panel is shown in **Table 1**. After antibody staining (10 min at RT in PBS supplemented with 4% FCS) cells were washed and resuspended with PBS supplemented with 4% FCS (both Merck, Darmstadt, Germany). Prior to flow cytometric analysis 7-AAD and Flow-Count Fluorospheres (both Beckman Coulter) were added to the samples for dead cell discrimination and single platform cell quantification, respectively. Flow cytometric analysis was performed with the Navios flow cytometer (Navios 3L 10C, Software 1.3, Beckman Coulter). For gating strategies see **Supplement Figures S6, 7.**

### Cell Lines and Cell Culture

The cell lines 293T, HT1080, HT1080-GD_2_ and SH-SY5Y were cultivated as recently described (26). NK-92 cells (human natural killer lymphoma #ACC 488; DSMZ, Braunschweig, Germany) were cultivated in RPMI 1640 medium (Lonza, Basel, Switzerland) supplemented with 10% FBS, 2 mM L-glutamine (c.c.pro, Oberdorla, Germany) and 400 IU/mL human IL-2 (Proleukin S, Novartis Pharma GmbH, Nürnberg, Germany). THP-1 (human acute monocytic leukemia, #ACC 16; DSMZ, Braunschweig, Germany) cells were cultivated in RPMI 1640 medium with 2 mM L-glutamine, 2-mercaptoethanol to a final concentration of 0.05 mM, 10% (v/v) heat-inactivated fetal bovine serum (HI-FBS), and 50 IU/ml penicillin and 50 µg/ml streptomycin. All cells were tested for mycoplasma contamination on a regular basis using the MycoAlert™ Mycoplasma Detection Kit (Lonza, Basel, Switzerland) according to the manufacturer’s protocol.

### Laboratory Manufacturing of IL-18 TRUCKs Targeting GD_2_ (Laboratory-Scale Process)

CD4^+^ and CD8^+^ T cells isolated by the CliniMACS Prodigy^®^ (see clinical-scale manufacturing) were transduced and expanded as previously described (34). Briefly, they were activated with anti-CD3/CD28 beads (Thermo Fisher Scientific, Waltham, MA, USA) at a ratio of 1:1 in TexMACS™ (Miltenyi Biotec) with 3% human serum (c.c.pro, Oberdorla, Germany) supplemented with 12.5 ng/ml IL-7 and IL-15 (PeproTech, Rocky Hill, NJ, USA). On the following day, T cells were either left untransduced or transduced with lentiviral particles by spinoculation using an MOI of 7 and addition of 5 µg/ml Polybrene Infection/Transfection Reagent (Merck Millipore, Burlington, MA, USA). The anti-CD3/CD28 beads were removed on the following day and cells were further cultivated in TexMACS™ medium supplemented with 3% human serum, 12.5 ng/ml IL-7 and IL-15 and splitting 1:2 every 2 - 3 days for a total expansion time of 12 days.

### Cryopreservation and Thawing of IL-18 TRUCKs Targeting GD_2_

Untransduced and transduced T cells were cryopreserved in Composol^®^ PS Fresenius Kabi, Bad Homburg, Germany/2.86% (w/v) HSA (Biotest, Dreieich, Germany) (D1) and SSP+ (Maco Pharma International GmbH, Langen, Germany) supplemented with 3.33% (w/v) HSA (D2), respectively after manufacturing by adjusting the cell counts and addition of DMSO (CryoSure-DMSO, USP grade; WAK Chemie, Steinbach, Germany) to a final concentration of 10% (v/v). After cryopreservation in a < -80°C freezer overnight, the cells were stored in the vapor phase above liquid nitrogen at < -140°C. For T cell phenotype analysis cells were thawed in RPMI 1640 medium with 20% (v/v) FCS (both Merck, Darmstadt, Germany) and rested for 1 h in RPMI 1640 with 10% FCS (37°C, 5% (v/v) CO_2_) before flow cytometric analysis. For functional analysis of the cryopreserved cells, they were thawed and seeded in TexMACS™ medium in a cell density of 2.5 x 10^6^ cells/ml and rested overnight.

### Co-Culture of Laboratory- and Clinical-Scale IL-18 TRUCKs With Target Cells

Directly after expansion or after cryopreservation and thawing (cryo), functionality of the laboratory- and clinical-scale IL-18 TRUCKs in comparison to untransduced T cells was assessed by co-culturing them with target cells. For flow cytometry and soluble mediator measurements, 5 x 10^4^ target cells were seeded in 800 µl of their respective culture medium, which was removed after 4 - 24 h followed by addition of effector cells according to the specified effector-to-target (E:T) ratio in 800 µl CTL medium. For cytotoxicity, microscopy and intracellular cytokine assessment, 2 x 10^4^ target cells were seeded in 200µl and co-cultured with effector cells accordingly.

### Flow Cytometry of Laboratory-Scale Experiments

The antibodies used for flow cytometric analysis are listed in **Supplement Table S1**. The transduction efficiency was analyzed with the Ganglidiomab-PE mAb. Intracellular staining of TNF-α was performed by using the IntraPrep Permeabilization Reagent (Beckman Coulter, Brea, CA, USA) according to the manufacturer’s instructions. Samples were read on a BD FACSCanto™ Flow Cytometer (Becton Dickinson, Franklin Lakes, NJ, USA). To determine cytotoxicity of engineered T cells, target cells were gated as CD3^-^ cells. Percentage of killed cells was normalized to untransduced cell co-cultures using the following formula:


$$\text{CD}3^{-}\text{ cell killing}=100\%-\frac{\text{CD}3^{-}\;cell\text{ frequency }(\text{co-culture})}{\text{CD}3^{-}\;cell\text{ frequency }(\text{respoective co-culture with untransduced T cells})}$$

### Multiplex Cytokine Analysis

Cytokine concentrations in the supernatant were determined using a customized LEGENDplex™ Multi-Analyte Flow Assay (BioLegend, San Diego, CA, USA), which allowed for the detection of human IL-2, IL-4, IL-10, IL-18, granzyme B, perforin, interferon (IFN)-γ, and tumor necrosis factor (TNF)-α. Samples were analyzed with LEGENDplex v8.0 software (BioLegend, San Diego, CA, USA).

### Determination of Cytotoxicity by LDH Assay

The release of lactate dehydrogenase (LDH) into the cell culture supernatant was assessed by using the Cytotoxicity Detection Kit (Roche, Basel, Switzerland). Cells lysed by adding Triton X-100 (Merck, Darmstadt, Germany) to a final concentration of 1% to all control wells served as maximum controls. Absorbance was assessed at a wavelength of 490 nm with a reference wavelength of 690 nm on a Synergy 2 Multi-Mode Microplate Reader (Biotek, Winooski, VT, USA). LDH release (%) was calculated according to the manufacturer’s protocol.

### Microscopy

Transmitted-light microscope images of co-cultures of target and effector cells were taken with an Olympus IX81 microscope combined with a digital B/W camera using 10x objective lenses and analyzed with Xcellence Pro image software (all from Olympus, Hamburg, Germany). Representative pictures are shown.

### XCelligence

Target cell killing by cryopreserved and thawed TRUCKs was furthermore determined with the XCelligence RTCA S16 Real Time Cell Analyzer and using E-Plates 16 PET (both ACEA Biosciences, San Diego, CA, USA). Background impedance of all wells was assessed with cell culture medium measurements. Afterwards, target cells were seeded in an amount of 1 x 10^5^ cells (SH-SY5Y) or 2 x 10^4^ cells (HT1080, HT1080-GD_2_) in 200 µl of their respective culture medium and adhesion was checked by measurements every 30 min. T cells were added after target cell adhesion shown by a constant impedance after 19 – 25 h. For this, 150 µl medium were carefully removed and replaced by T cells in the specified E:T-ratios in 150 µl CTL medium. Impedance was measured every 30 min. Cell indices were normalized to the respective indices after T cell addition.

### Cell Migration Assay

To test the chemo-attractive potential of supernatants that were derived from co-culture experiments of primary human T cells transduced with the IL-18 TRUCK vector and ${\text{GD}}_{2}^{+}$ target cells, migration assays with THP-1 or NK-92 cells were performed using a Boyden chamber (NeuroProbe, Gaithersburg, MD, USA) as recently described (26). The cell number was calculated using an Olympus IX71 microscope and imageJ 1.53k software. To normalize results from different plates, cell numbers of migrated cells towards untransduced T cells as background migration were subtracted from all values for each plate.

### Isolation of Genomic DNA and Determination of the VCN by qPCR

Genomic DNA was isolated from 1 x 10^6^ transduced or untransduced cells (-20°C frozen cell pellets) with the QIAamp^®^ DNA blood mini kit (Qiagen, Hilden, Germany) according to the manufacturer’s protocol. The determination of the VCN by qPCR was performed as recently described (35).

### Statistics

Statistical analysis was performed with GraphPad Prism V.9.1.2 using the Kruskal-Wallis and uncorrected Dunn’s test. Only mean ranks of preselected data sets were compared: large-scale TRUCKs cultured alone or co-cultured with different target cells, large-scale TRUCKs co-cultured with different target cells immediately after generation (indicated by black asterisks) or cryopreservation (indicated by grey asterisks), large-scale TRUCKs co-cultured with different target cells in comparison to the same co-cultures with all laboratory-scale manufactured cells. Significant differences are shown (*p ≤ 0.05, **p ≤ 0.01).

## Results

### GMP-Compliant Manufacturing Process of IL-18 TRUCKs Targeting GD_2_ Using Automated Cell Processing in a Closed System

To assess feasibility, we performed two complete GMP-compliant processes (D1 and D2 with two individual donor lymphocyte products) to manufacture IL-18 TRUCKs targeting GD_2_ using an automated TCT protocol for CliniMACS Prodigy^®^ as shown in **Figure 1**.

### Recovery and Purity of CD4^+^ and CD8^+^ Cells Enriched in an Automated Process

**Figure 2:**
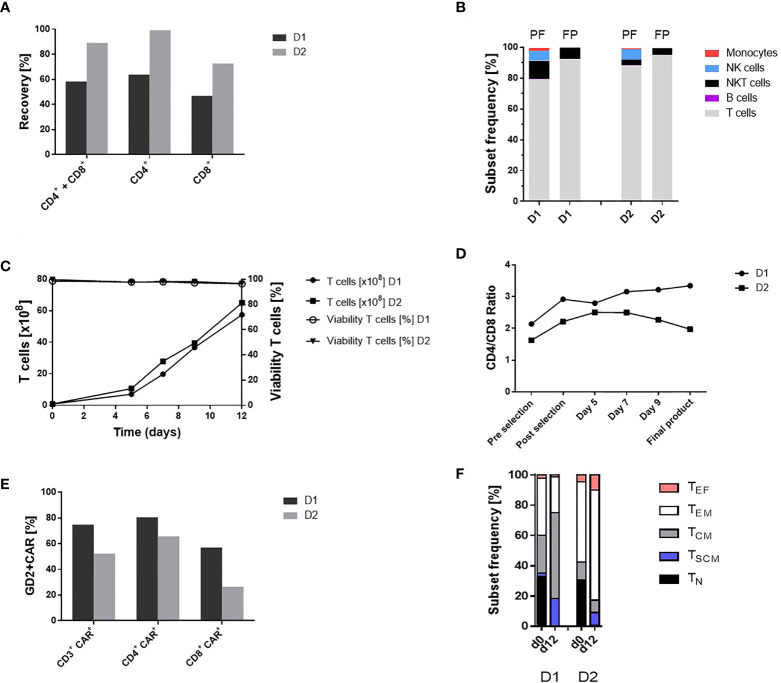
Automated GMP-compliant manufacturing of IL-18 TRUCKs targeting GD_2_ using the CliniMACS Prodigy^®^ (clinical-scale process). **(A)** Recovery (cell population in percent of cell population pre-enrichment) of CD4^+^ and CD8^+^ cells after enrichment. **(B)** Cell composition after enrichment of CD4^+^ and CD8^+^ cells and in the final products. The achieved T cell purity in the positive fraction (PF) was 79.1% D1 and 87.7% D2 with contaminating CD56^+^CD3^+^ NKT cells (12.0% D1 and 3.8% D2), CD56^+^CD3^-^ NK cells (6.4% D1 and 6.8% D2), CD14^+^ monocytes (1.9% D1 and 0.6% D2) and CD20^+^ B cells (0.2% D1 and 0.5% D2). Cell composition of the final products (FP) with a purity of 92.1% D1 and 95.0% D2 T cells (CD3^+^/CD56^-^) and impurities with NKT cells (7.6% D1 and 4.4% D2) as well as residual 0.1% D1 and 0.2% D2 NK cells. Residual B cells (0.05% D2) were detected but no monocytes in the final products. **(C)** Expansion and viability of T cells (CD3^+^ CD56^-^) during 12-day manufacturing. **(D)** CD4/CD8 ratio during cultivation. The CD4/CD8 ratio changed during the manufacturing process (post-enrichment) from 2.9 to 3.3 D1 and 2.2 to 2.0 D2 in the final products. **(E)** Transduction rate of CD3^+^ cells and CD4^+^ CD8^+^ subtypes in the final products. **(F)** T cell phenotypic analysis of the starting material on day 0 (lymphapheresis) and in the final products on day 12. The analyses were performed by flow cytometry based on the expression of CD45RO, CCR7 and CD95 among viable CD3^+^ cells to define naïve (T_N_: CD45RO^-^, CCR7^+^, CD95^-^), stem-cell memory (T_SCM_: CD45RO^-^, CCR7^+^, CD95^+^), central memory (T_CM_: CD45RO^+^, CCR7^+^ CD95^+^), effector memory (T_EM_: CD45RO^+^, CCR7^-^ CD95^+^) and effector (T_EF_: CD45RO^-^, CCR7^-^ CD95^+^) T cell subsets. T_N_ (33.1% D1 and 30.0% D2), T_CM_ (25.1% and 11.8% D2) and T_EM_ (37.6% D1 and 53.0% D2) cells were present in the initial product with differences in the T cell subsets (T_SCM_: 2.2% D1 and 0.62% D2, T_EF_: 2.1% D1 and 4.62% D2). In contrast, the final TRUCK products harbored cells with less differentiated memory phenotypes with a T_CM_ (56.9% D1 and 8.17% D2) and T_SCM_ (18.3% D1 and 8.62% D2) cells, T_EM_ (23.6% D1 and 72.74% D2) and T_EF_ (1.2% D1 and 9.9% D2) cells and the decrease of T_N_ (<0.1% D1 and 0.54% D2) cells For cell composition, cells **(B, D)** were gated as viable CD45^+^ cells using lineage-specific markers: T cells (CD3^+^ CD56^-^), monocytes (CD14^+^), NK cells (CD56^+^ CD16^+^), NKT cells (CD56^+^ CD3^+^), B cells (CD20^+^). n = 2.

Starting with the lymphapheresis, 1.14 x 10^9^ D1 and 1.17 x 10^9^ D2, respectively, CD4^+^ and CD8^+^ cells (20.6% D1 and 46.1% D2 of unstimulated short time lymphapheresis) were applied for enrichment. An amount of 0.67 x 10^9^ D1 and 0.88 x 10^9^ D2 CD4^+^ and CD8^+^ cells were obtained representing a recovery of 58.8% D1 and 75.2% D2. Thereby, the isolation of CD4^+^ cells with a recovery of 63.9% D1 and 84.04% D2 was more effective than enrichment of CD8^+^ cells with 46.8% D1 and 61.7% D2 recovery, respectively (**Figure 2A**). The achieved T cell purities and cell compositions are shown in **Figure 2B**. Due to the CD8 based isolation procedure, NK as well as NKT cells were not completely depleted releasing 7.6% D1 and 4.4% D2 contaminating NKT cells in the final cell product (**Figure 2B**). The CD4/CD8 ratio pre-enrichment was 2.1 D1 and 1.6 D2 and post-enrichment 2.9 D1 and 2.2 D2 reflecting a superior recovery of CD4^+^ cells (**Figure 2D**).

### Expanded IL-18 TRUCKs Targeting GD_2_ Display a High Purity and Favorable Phenotype

In accordance with the TCT protocol, the cell expansion started with 0.84 x 10^8^ D1 and 0.9 x 10^8^ D2 viable T cells (corresponding 1.18 x 10^8^ D1 and 1.0 x 10^8^ D2 viable WBC) after enrichment of CD4^+^ and CD8^+^ cells. The expansion rate during the 12-day manufacturing process was 68.3-fold D1 and 71.4-fold D2 for T cells. Viability of the cells was >96% during the whole process until final harvest (**Figure 2C**). Cell concentration increased in a constant culture volume from 3.8 x 10^6^/ml D1 on day 5 and 5.5 x 10^6^/ml D2 to 31.2 x 10^6^/ml D1 and 34.2 x 10^6^/ml D2 in the final products (**Figure S1A**). Monitoring of cell culture condition revealed a pH of 7.0 - 7.1 and a glucose level above the critical level of 100 mg/dl. (**Figure S1B**). T cell purity and cell composition of the final products is shown in **Figure 2B**. During expansion, the CD4/CD8 ratio increased in the first run from 2.9 to 3.3 D1 but decreased slightly in the second run from 2.2 to 2.0 D2 (**Figure 2D**). The proportion of T cell phenotypes during *ex vivo* cell expansion was analyzed by comparing T cell phenotypes in the initial product post CD4^+^ and CD8^+^ enrichment at process day 0 and in the final product at process day 12 as shown in **Figure 2F**.

### Transduction of T Cells With the “All-In-One” Lentiviral Vector Is Highly Efficient

The recently described “all-in-one” lentiviral vector (26) encoding constitutive GD_2_ CAR and inducible IL-18 expression was used for cell transduction on day 1 of the manufacturing process with a multiplicity of infection (MOI) of 29 for D1 and 10 for D2 derived T cells. The percentage of transduced CD3^+^ cells in the final product, was 74.9% D1 and 52.2% D2, while CD4^+^ cells exhibited a higher transduction efficiency (80.6% D1 and 65.8% D2) compared to CD8^+^ cells (57.2% D1 and 26.5% D2) (**Figure 2E**). The VCN determined by qPCR of genomic DNA in the final products were 2.6 D1 and 2.4 D2 copies/cell, respectively.

### Preclinical *In Vitro* Characterization Using Clinical- and Laboratory-Scale Manufactured TRUCKs

**Figure 3:**
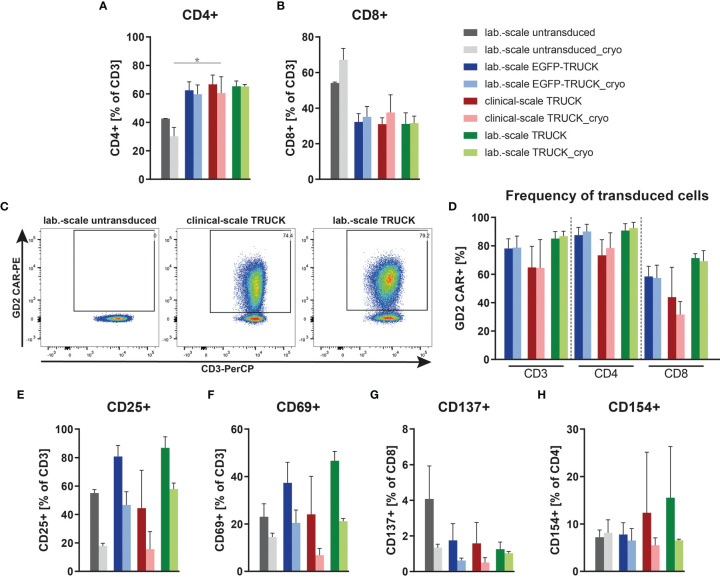
Clinical-scale-manufactured IL-18 TRUCKs targeting GD_2_ are transduced and pre-activated at similar efficacies compared to laboratory-scale-manufactured TRUCKs. IL-18 TRUCKs targeting GD_2_ were generated using the CliniMACS Prodigy^®^ (clinical-scale TRUCK; n=2) or under laboratory conditions (lab.-scale TRUCK; n=3). Untransduced T cells (lab.-scale untransduced; n=3) as well as GD_2_ TRUCKs with inducible EGFP expression (EGFP-TRUCK; n=3) served as control. The manufactured cells were either characterized directly after the generation process (d12) or after cryopreservation and thawing (cryo). **(A, B)** Frequency of **(A)** CD4^+^ and **(B)** CD8^+^ T cells in the final cell product. **(C, D)** Percentage of CAR^+^ cells of **(C)** CD3^+^ cells shown as representative plots and **(D)** CD3^+^, CD4^+^ and CD8^+^ cells as assessed by staining of the scFv-domain of TRUCKs with a Ganglidiomab antibody after expansion. **(E–H)** Expression of the activation markers **(E)** CD25 on CD3^+^, **(F)** CD69 on CD3^+^, **(G)** CD137 on CD8^+^ and **(H)** CD154 on CD4^+^ T cells. **(A, B, D–H)** Data are shown as mean ± SD. Statistical differences of large-scale TRUCKs directly after generation or cryopreservation as well as in comparison to laboratory-scale manufactured cells were assessed by Kruskal-Wallis and Dunn’s test, significant differences are shown (*p ≤ 0.05).

IL-18 TRUCKs targeting GD_2_ manufactured using the CliniMACS Prodigy^®^ (referred to as clinical-scale TRUCKs) were compared to the respective TRUCKs generated under laboratory conditions (referred to as laboratory-scale TRUCKs). These cells were manufactured manually as previously described (34) using the same isolated CD4^+^ and CD8^+^ T cell starting population, lentiviral vector, expansion media, cytokines, and experimental timing. Untransduced T cells as well as GD_2_ TRUCKs with inducible EGFP expression (referred to as EGFP-TRUCKs) were generated in laboratory scale and served as controls. From a starting fraction of 0.6 x 10^6^ cells, laboratory-scale TRUCKs expanded to a cell number of 48 x 10^6^ cells, which was slightly lower compared to untransduced T cells and EGFP-TRUCKs with final cell numbers of 53 x 10^6^ and 54 x 10^6^ cells, respectively (**Figure S2A**). Both, laboratory- and clinical-scale IL-18 TRUCKs, showed an enhanced frequency of CD4^+^ T cells after expansion with CD4/CD8 ratios of 2.2 for clinical- and 2.1 for laboratory-scale TRUCKs, whereas it was 0.79 for untransduced T cells (**Figures 3A, B**). Utilizing the recently described “all-in-one” vector” (26), we obtained a high percentage of transduced cells with 65% and 85% under clinical scale as well as under laboratory conditions, respectively, with a higher frequency of transduced CD4^+^ T cells than CD8^+^ T cells (**Figures 3C, D**). Activation marker expression on clinical-scale TRUCKs, assessed after expansion of T cells with anti-CD3/CD28 stimulation and IL-7 and IL-15 supplementation, revealed CD25 and CD69 expression on CD3^+^ T cells (45% and 24%, respectively), moderate CD154 expression on CD4^+^ cells (13%) and absent CD137 expression on CD8^+^ cells (**Figures 3E–H**); similar data were obtained for laboratory-scale cells. Clinical use of modified cells often requires cryopreservation to allow for centralized manufacturing and administration flexibility (36). To assess the feasibility of cryopreservation of the obtained T cell product, the manufactured T cells were also characterized after freezing and thawing. Cryopreservation of clinical-scale IL-18 TRUCKs did not significantly change their activation state, CD4/CD8 ratios and frequency of transduced cells (**Figures 3A, B, D–H**).

### Clinical-Scale-Manufactured IL-18 TRUCKs Targeting GD_2_ Specifically Respond to ${\text{GD}}_{2}^{+}$ Target Cells With an Increase of Activation Marker Expression

**Figure 4:**
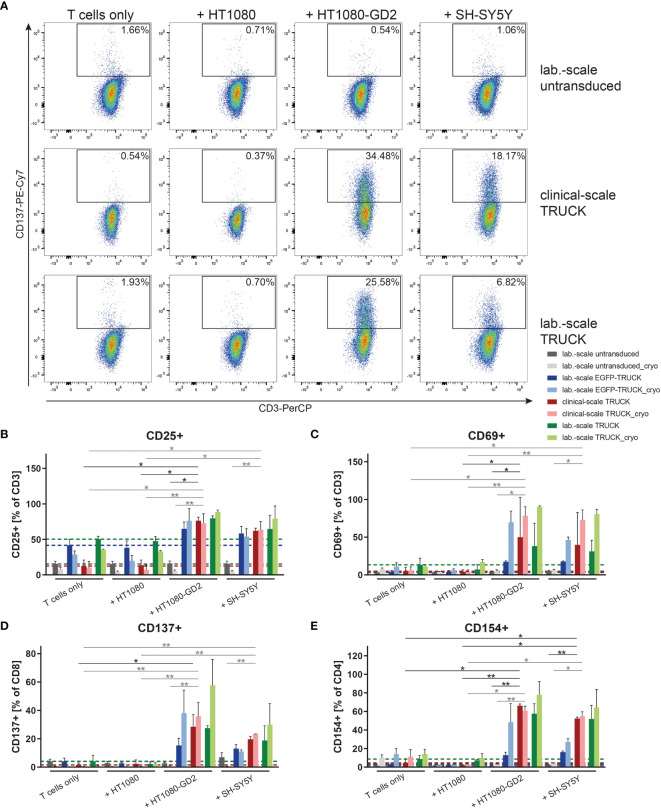
Clinical-scale-manufactured IL-18 TRUCKs targeting GD_2_ specifically respond to ${\text{GD}}_{2}^{+}$ target cells with an increase of activation marker expression. IL-18 TRUCKs targeting GD_2_ were generated using the CliniMACS Prodigy^®^ (clinical-scale TRUCK; n=2) or under laboratory conditions (lab.-scale TRUCK; n=3). Untransduced T cells (lab.-scale untransduced; n=3) as well as GD_2_ TRUCKs with inducible EGFP expression (EGFP-TRUCK; n=3) served as control. The manufactured cells were tested for GD_2_-CAR-mediated activation either directly after the generation process (d12) or after cryopreservation and thawing (cryo) by co-cultivation with the indicated target cells for 48h in an effector-to-target (E:T) ratio of 4:1 or cultivation alone (T cells only). **(A–E)** Frequency of **(A)** CD137^+^ of CD8^+^ as representative plots**, (B)** CD25^+^ of CD3^+^, **(C)** CD69^+^ of CD3^+^, **(D)** CD137^+^ of CD8^+^ and **(E)** CD154^+^ of CD4^+^ T cells as determined by flow cytometry. **(B–E)** A dashed line indicates background levels of the respective expression by untransduced T cells (grey), EGFP-TRUCKs (blue), as well as clinical-scale (red) and laboratory-scale (green) TRUCKs cultured alone. Data is shown as mean ± SD. Statistical differences of clinical-scale TRUCKs co-cultured with different target cells after generation or cryopreservation as well as in comparison to laboratory-scale manufactured cells were assessed by Kruskal-Wallis and Dunn’s test, significant differences are shown (*p ≤ 0.05, **p ≤ 0.01).

We tested the ability of the generated TRUCKs to recognize and react towards ${\text{GD}}_{2}^{+}$ target cells. Manufactured cells were co-cultivated either with GD_2_^-^ HT1080, HT1080 cells expressing GD_2_ or with the GD_2_^+^ neuroblastoma cell line SH-SY5Y as target cells and examined for activation marker expression. Clinical-scale IL-18 TRUCKs targeting GD_2_ responded with increased expression of CD25 and CD69 by 50 – 64% and 35 – 45%, respectively, on all CD3^+^ T cells to both GD_2_-expressing target cells, whereas no specific response towards unmodified HT1080 cells was detected (**Figures 4B, C** and **S3A–D**). The slightly lower activation response towards SH-SY5Y in comparison to HT1080-GD_2_ coincides with the lower GD_2_ expression levels (26). Clinical-scale compared to laboratory-scale TRUCKs exhibited a similar activation marker increase for all markers and target cells, and also cryopreserved TRUCKs did not show significantly different responses regarding expression of CD137 on CD8^+^ and CD154 on CD4^+^ T cells, the same effects were observed, indicating cells of the CD4^+^ and CD8^+^ populations in manufactured IL-18 TRUCKs were specifically and effectively activated in response to ${\text{GD}}_{2}^{+}$ target cells (**Figures 4A, D, E** and **S3E–H**). Assessment of respective activation marker expression levels as mean fluorescence intensity (MFI) or in further effector-to-target (E:T) ratios confirmed the observed results (**Figures S3A–H**).

### Clinical-Scale-Manufactured IL-18 TRUCKs Targeting GD_2_ Increased Release of Cytokines Upon Target Recognition

**Figure 5:**
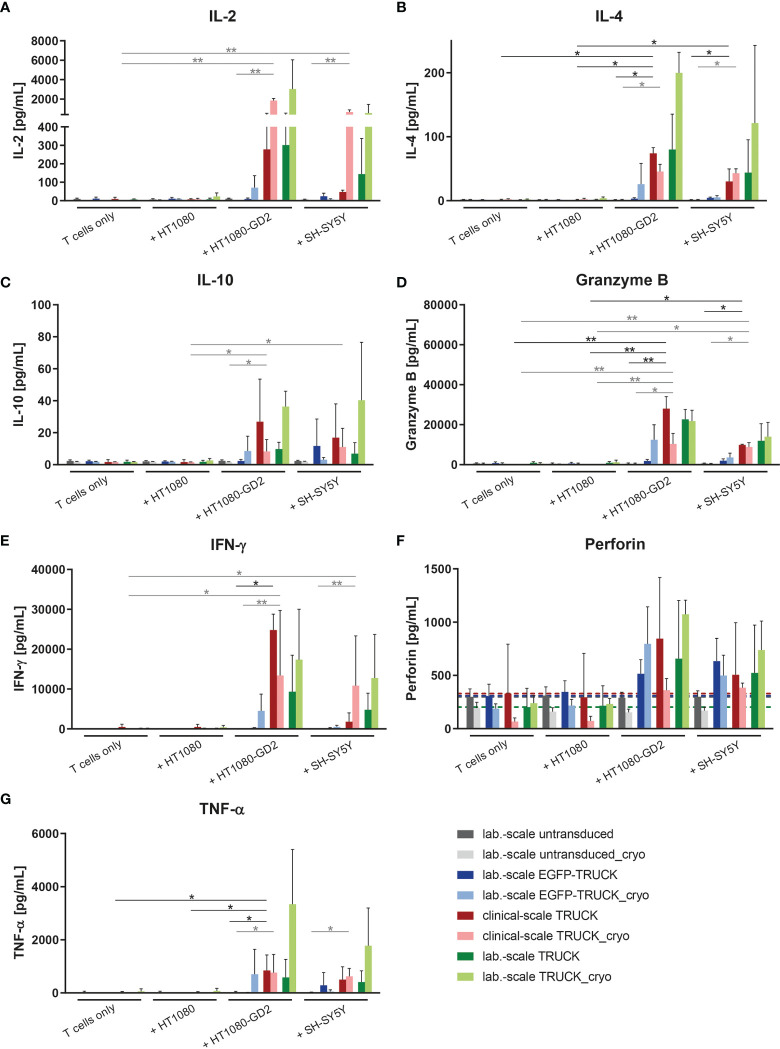
Clinical-scale-manufactured IL-18 TRUCKs targeting GD_2_ specifically react to target recognition with increased release of soluble mediators. IL-18 TRUCKs targeting GD_2_ were generated using the CliniMACS Prodigy^®^ (clinical-scale TRUCK; n=2) or under laboratory conditions (lab.-scale TRUCK; n=3). Untransduced T cells (lab.-scale untransduced; n=3) as well as GD_2_ TRUCKs with inducible EGFP expression (EGFP-TRUCK; n=3) served as control. The manufactured cells were tested for functionality either directly after the generation process (d12) or after cryopreservation and thawing (cryo) by co-cultivation with the indicated target cells in an effector-to-target (E:T) ratio of 8:1 or cultivation of T cells only. The concentration of released cytokines **(A)** IL-2, **(B)** IL-4, **(C)** IL-10, **(D)** granzyme B, **(E)** IFN-γ, **(F)** perforin, and **(G)** TNF-α in the cell culture supernatants after 48 h was assessed by LEGENDPlex™. **(F)** A dashed line indicates background levels of the respective cytokine release by untransduced T cells (grey), EGFP-TRUCKs (blue), as well as clinical-scale (red) and laboratory-scale (green) TRUCKs cultured alone. **(A–G)** Data are shown as mean ± SD. Statistical differences of clinical-scale TRUCKs co-cultured with different target cells after generation or cryopreservation as well as in comparison to all laboratory-scale manufactured cells were assessed by Kruskal-Wallis and Dunn’s test, significant differences are shown (*p ≤ 0.05, **p ≤ 0.01).

The release of cytokines by manufactured cells into the cell co-culture supernatants upon target contact was assessed. In co-cultures with HT1080-GD_2_ cells and, to a lower extent also with SH-SY5Y cells, the release of several cytokines and cytolytic factors was upregulated in clinical-scale IL-18 TRUCKs compared to background secretion: IL-2 (108 and 25-fold), IL-4 (39 and 23-fold), IL-10 (5.8 and 2.2-fold), interferon (IFN)-γ (70 and 49-fold), granzyme B (290 and 150-fold), perforin (3.7 and 2.7-fold) and tumor necrosis factor (TNF)-α (430 and 380-fold; all in an E:T ratio of 8:1) (**Figures 5** and **S4**). Thereby, antigen-induced cytokine release by clinical-scale TRUCKs was similar compared to laboratory-scale TRUCKs and, furthermore, cryopreservation of cells did not significantly change the secretion pattern. A higher expression of TNF-α, by IL-18 TRUCKs upon specific target contact was confirmed by intracellular staining (**Figure S3I**).

### Clinical-Scale-Manufactured IL-18 TRUCKs Targeting GD_2_ Specifically Eliminate ${\text{GD}}_{2}^{+}$ Target Cells

**Figure 6:**
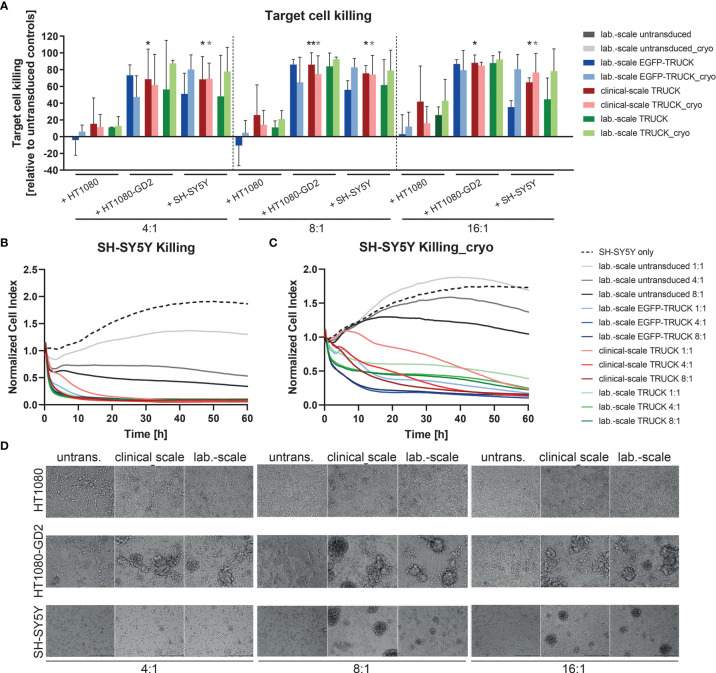
Clinical-scale-manufactured IL-18 TRUCKs targeting GD_2_ specifically eliminate ${\text{GD}}_{2}^{+}$ target cells. IL-18 TRUCKs targeting GD_2_ were generated using the CliniMACS Prodigy^®^ (clinical-scale TRUCK; n=2) or under laboratory conditions (lab.-scale TRUCK; n=3). Untransduced T cells (lab.-scale untransduced; n=3) as well as GD_2_ TRUCKs with inducible EGFP expression (EGFP-TRUCK; n=3) served as control. The manufactured cells were tested for cytotoxicity either directly after the generation process (d12) or after cryopreservation and thawing (cryo) by co-cultivation with the indicated target cells and in the indicated effector-to-target (E:T) ratios. **(A)** Target cell killing after 48h was measured by flow cytometry as percentage of killed CD3^-^ cells relative to those eliminated in co-cultures with untransduced T cells. Data is shown as mean ± SD. Statistical differences of large-scale TRUCKs co-cultured with different target cells directly after generation or cryopreservation as well as in comparison to all laboratory-scale manufactured cells were assessed by Kruskal-Wallis and Dunn’s test, whereby only significant differences are shown (*p ≤ 0.05, **p ≤ 0.01). **(B, C)** Killing of SH-SY5Y cells by the generated cells **(B)** directly after their generation (here: lab.-scale untransduced n=2, clinical-scale TRUCK n=1) or **(C)** after cryopreservation and thawing was determined with the XCelligence Real-Time Cell Analyzer. Cell indices were normalized to the respective indices after T cell addition. Data is shown as mean. **(D)** Representative transmitted-light microscope images of co-cultures of fresh effector cells with target cells taken after 48 h by an Olympus IX81 microscope combined with a digital B/W camera using 10x objective lenses.

Finally, the killing capacity of manufactured IL-18 TRUCKs targeting GD_2_ was determined by flow cytometric analysis of target cells in the co-cultures. Relative to co-cultures of untransduced T cells with HT1080-GD_2_ and SH-SY5Y, clinical-scale TRUCKs eliminated 69 - 88% of HT1080-GD_2_ and 65 - 86% of SH-SY5Y cells at different E:T ratios, whereas the frequency of HT1080 in co-cultures with the clinical-scale TRUCKs was on the level of untransduced T cells or laboratory-scale manufactured cells, likely representing expected allo-reactivity (**Figure 6A**). Compared to TRUCKs generated in the laboratory-scale, clinical-scale-manufactured TRUCKs exhibited a similar cytotoxic ability to eliminate ${\text{GD}}_{2}^{+}$ target cells, which was moreover not impeded by cryopreservation. The release of lactate dehydrogenase (LDH) into the supernatant as parameter for cytolysis confirmed the result; cytotoxicity was enhanced in co-cultures of both IL-18 TRUCKs with HT1080-GD_2_ cells (18-23% and 0-17% for large- and laboratory-scale TRUCKs, respectively) compared to the respective co-cultures with unmodified HT1080 cells, in which LDH was not released above background of cells cultured alone (**Figure S5A**). Target cell death by LDH release in co-cultures of clinical-scale TRUCKs with SH-SY5Y was also enhanced (6-16%) and similar to the cytotoxicity by laboratory-scale TRUCKs (2-8%). LDH measurements in the co-cultures with cryopreserved T cells revealed a similar cytotoxic capability towards ${\text{GD}}_{2}^{+}$ target cells. Real-time measurement of target cell viability confirmed these results. After addition of both TRUCKs to adherent SH-SY5Y cells, the cell index was rapidly reduced, resulting in almost complete absence of adherent target cells after co-cultivation with laboratory- or clinical-scale TRUCKs for 60 h in different ratios (**Figures 6B, C**). Transmitted-light microscopy visualized the process of target cell elimination. In co-cultures of HT1080 with all T cell products, cells are distributed equally, and the target cells stayed viable in all E:T ratios (**Figure 6D**). In co-cultures of both TRUCKs with HT1080-GD_2_ or SH-SY5Y, the target cells were diminished or even absent after 72 h and T cells formed large clusters around the target cells. For thawed TRUCKs, the clusters tended to be even larger and already appeared at low E:T ratios in co-cultures with SH-SY5Y cells (**Figure S5B**).

### Clinical-Scale-Manufactured IL-18 TRUCKs Targeting GD_2_ Released IL-18 in a Target-Specific Manner Leading to Innate Immune Cell Attraction

**Figure 7:**
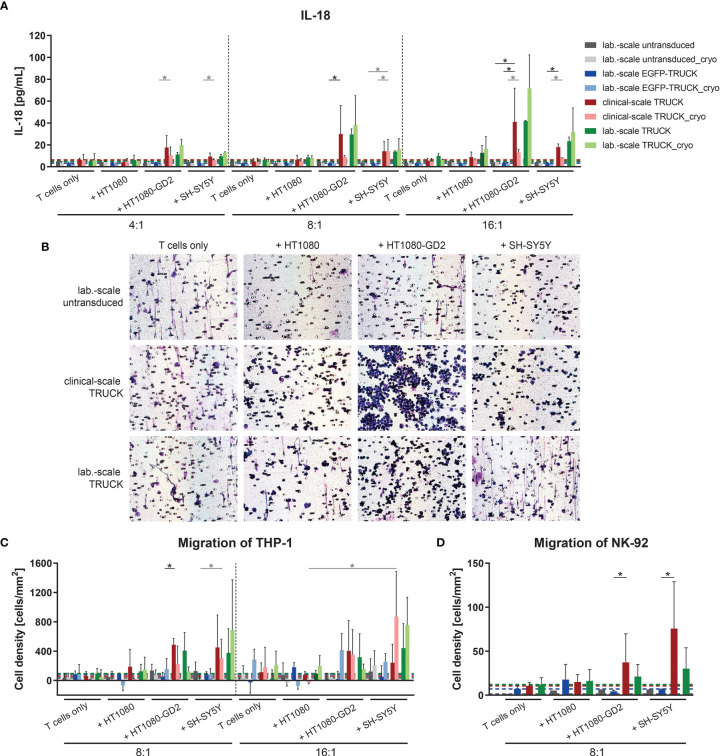
Clinical-scale-manufactured IL-18 TRUCKs targeting GD_2_ released IL-18 in a target-specific manner leading to innate immune cell attraction. IL-18 TRUCKs targeting GD_2_ were generated using the CliniMACS Prodigy^®^ (clinical-scale TRUCK; n=2) or under laboratory conditions (lab.-scale TRUCK; n=3). Untransduced T cells (lab-scale untransduced; n=3) as well as GD_2_ TRUCKs with inducible EGFP expression (EGFP-TRUCK; n=3 [except for D, in which n=1]) served as control. The manufactured cells were tested for functionality either directly after the generation process (d12) or after cryopreservation and thawing (cryo) by co-cultivation with the indicated target cells in the indicated effector-to-target (E:T) ratios or cultivation alone (T cells only). **(A)** The concentration of released cytokines in the cell culture supernatants after 48h was assessed by LEGENDPlex™. The chemoattractive potential of IL-18 released by TRUCKs upon target recognition in terms of the migration of **(B, C)** THP-1 and **(D)** NK-92 cells was assessed. Supernatant of engineered T cells cultured alone (T cells only) or together with the indicated target cells for 48 h was placed in a Boyden chamber, covered with an 8 µm polycarbonate membrane and incubated for another 4h. Medium served as the control supernatant. Cells migrated through the membranes were Giemsa stained. **(B)** Representative pictures of Giemsa stained THP-1 cells (violet) on the bottom of the membrane. **(C, D)** The number of cells that migrated through the membrane was determined. To normalize results from different plates, cell numbers of migrated cells towards untransduced T cells only were subtracted from all values. **(A, C, D)** Data is shown as mean ± SD. A dashed line indicates background levels of the respective cytokine release by untransduced T cells (grey), EGFP-TRUCKs (blue), as well as clinical-scale (red) and laboratory-scale (green) TRUCKs cultured alone. Statistical differences of large-scale TRUCKs co-cultured with different target cells after generation or cryopreservation as well as in comparison to laboratory-scale manufactured cells were assessed by Kruskal-Wallis and Dunn’s test, significant differences are shown (*p ≤ 0.05).

To address the ability of the generated TRUCKs to selectively release IL-18 following CAR engagement of GD_2_ target antigen, the cytokine was measured in the co-culture supernatants. Importantly, the release of IL-18 into the cell culture supernatant by freshly-generated or cryopreserved clinical-scale TRUCKs was induced in a target-specific manner up to 41 pg/ml upon HT1080-GD_2_ and 18 pg/ml upon SH-SY5Y encounter (**Figure 7A**). To assess the effect of anti-GD_2_ IL-18 TRUCK-induced cytokines with respect to the recruitment of innate immune cells, we used a modified Boyden chamber assay to compare the migration potential of cell supernatants collected from co-culture experiments of IL-18 TRUCKs vs. untransduced T cells and ${\text{GD}}_{2}^{+}$ target cells (HT1080-GD_2_ and SH-SY5Y) to promote the recruitment of monocytes (THP-1 cells) and NK cells (NK-92 cells). The capacity of IL-18 containing supernatants to recruit innate immune cells was shown by positive Giemsa staining of the transwell membrane through which migrating cells were recruited (**Figure 7B**). Giemsa staining of migrated cells revealed that increased numbers of the monocytic cells THP-1 as well as NK-92 cells were recruited by supernatants collected from IL-18 TRUCKs co-cultured with HT1080-GD_2_ or SH-SY5Y target cells compared to untransduced T cells, EGFP-TRUCKs or upon co-culture with ${\text{GD}}_{2}^{‐}$ target cells, likely as a result of induced IL-18 cytokine secretion (**Figures 7C, D**). Taken together, supernatants from TRUCKs releasing IL-18 in an inducible manner by CAR activation upon antigen recognition exhibit an innate immune cell recruitment potential *in vitro* indicating IL-18 biological activity.

## Discussion

In general, CAR and TRUCK vector design is a critical aspect for the generation of engineered T cells. Especially, the costimulatory domains within the CAR might be of high interest to improve CAR T-cell activity and long-lasting persistence with reduced T cell exhaustion, also with respect to the targeted tumor antigen (37). TRUCKs additionally combine the redirected CAR T cell attack with the on-site release of a biologically active protein while avoiding its systemic toxicity, thereby holding promise to modulate the environment of the targeted solid tumor. The cytokine of choice within the TRUCK concept should be chosen with respect to the desired immune response within the tumor microenvironment (TME). The IL-18 cytokine creates a proinflammatory environment, recruits bystander effector cells to the tumor site and enhances cytolytic activity (4–6). Therefore, IL-18 was an attractive cytokine for the development of the “all-in-one” lentiviral vector combining constitutive anti-GD_2_ CAR expression and inducible IL-18 (26) as well as for the automated and closed processing for GMP-compliant manufacturing process for CAR T cells (24, 25).

TRUCKs combine the redirected CAR T cell attack with the on-site release of a biologically active protein while avoiding its systemic toxicity, thereby holding promise to modulate the environment of the targeted solid tumor. Further development of the strategy is based on design and broad *in vitro* characterization of the “all-in-one” lentiviral vector combining constitutive anti-GD_2_ CAR expression and inducible IL-18 (26) as well as on an automated and closed processing for GMP-compliant manufacturing process for CAR T cells (24, 25). We present the proof of feasibility for translation of the method to activate and expand IL-18 TRUCKs targeting GD_2_ for clinical application.

Our protocol is optimized to produce CAR-engineered T cells in clinically sufficient numbers under GMP-compliance using the CliniMACS Prodigy^®^ platform that integrates different steps of manufacturing including cell isolation, activation, transduction, cell washing, cultivation and formulation of the final product in a single process and thus minimizes variability emanating from various manual work steps. The fully integrated modular system allows for flexibility and standardized procedure at the same time, which is the key for the production of personalized cell products of various kinds. Multiple steps are required to produce gene modified effector cells starting with enrichment of CD4^+^ and CD8^+^ T cell subset followed by activation, transduction and expansion of effector cells. CD4^+^ and CD8^+^ enrichment is regarded as a safety procedure to decrease blast counts in the culture (38). CD4^+^ and CD8^+^ enrichment also decreases contaminating cells such as monocytes, which inhibit CAR T cell expansion (39). The primary objective was the feasibility of cell production for 3 dose levels (e. g. 5 × 10^5^, 1 ×10^6^, and 3 × 10^6^ anti-GD_2_ IL-18 TRUCKs/kg). For enrichment of CD4^+^ and CD8^+^ cells the TCT process is limited to a maximum of 3 x 10^9^ target cells. This means for our two processes that only a part (20.6% D1 and 46.1% D2) of the lymphapheresis from a healthy donor was used for enrichment. Likewise, only part of enriched cells (12.5% D1 and 10.0% D2) could be applied for activation and expansion. Due to the limited culture volume and growth area, it is recommended to start with 1 x 10^8^ cells. Any remainder may be frozen as backup for the patient.

After CD4^+^ and CD8^+^ enrichment we found high T cell purities with low contaminating cell populations of CD8^+^ NK and NKT cells which are not removed during the CD4^+^ and CD8^+^ enrichment step also described by other groups (23, 38). With a T cell expansion rate of 68.3-fold D1 and 71.4-fold D2 and a transduction rate of 77.7% D1 and 55.1% D2 we reached sufficient cell doses of 4.8 x 10^9^ D1 and 3.7 x 10^9^ D2 IL-18 TRUCKs. This would allow for the application of 60.0 x 10^6^ cells/kg respectively 46.0 x 10^6^ cells/kg IL-18 TRUCKs targeting GD_2_ to a recipient weighing 80 kg even in a multi-dose base. We and others show the eligibility of the T cell transduction (TCT) process developed by Miltenyi Biotec with fixed process parts (enrichment, activation and transduction) as well as the possibility of variable culture set up by modular programming of the activity matrix (22–25, 38, 40–43).

Safety aspects in the clinical use of Advanced Therapy Medicinal Products (ATMPs) have high priority. Lentiviral vectors are known to have a lower risk for mutational oncogenesis than γ-retroviral vectors (38). The European Medicines Agency (28) reflection paper (EMA/CAT/190186/2012) on the management of clinical risks deriving from insertional mutagenesis highlighted the VCN as a risk factor for oncogenesis and recommended risk assessment and management of the integration copy numbers, integration profile and sites in cellular products. The IL-18 TRUCK final products contained 2.6 D1 and 2.4 D2 copies/cell, respectively, which is below 5 copies/cell that is considered to be safe (44). The transduction rate of 74.9% D1 and 52.2% D2 in the final products was higher than reported by other groups using CliniMACS Prodigy^®^ for production of CAR T cells (22, 23, 25, 38, 40–43, 45). Transduction efficiency was higher for CD4^+^ compared to CD8^+^ T cells as also shown by previous reports (25, 43).

To prolong the *in vivo* persistence of CAR T cells in patients, enrichment of cell products with less differentiated T cell subsets such as central memory (T_CM_) or stem cell memory T (T_SCM_) T cells is thought to be crucial. These subpopulations have gained substantial attention, as the adoptive transfer of even low numbers of T cells from these subsets can reconstitute robust and long-term maintained immune responses (46, 47). IL-18 TRUCK final product T cells from Donor 1 showed a T_cm_/T_scm_ phenotype while Donor 2 showed predominantly a T_EM_ phenotype. CD3/CD28 activation and culture of naïve T (48) cells in presence of IL-7 and IL-15 promotes the acquisition of T_CM_ or T_SCM_ phenotypes (22, 23, 38, 49). This is in line with previous reports (23, 43). In addition, whereas T cells expressing CARs with CD28 domains predominantly differentiate into effector memory T (T_EM_) cells, *in vitro* expansion of 4-1BB-containing CAR T cells as the TRUCKs used here produces a higher proportion of T_CM_ cells (50). We also demonstrate, that the TRUCK manufacturing process did not lead to unspecific secretion of IL-18 even after T cell activation with TransAct (CD3/CD28) and cytokines (IL-7 and IL-15).

*In vitro* characterization of the obtained engineered T cell product revealed an upregulation of activation markers (CD25, CD69, CD137 and CD154) on both CD4^+^ and CD8^+^ T cells and a variety of pro-inflammatory cytokines and cytotoxic mediators (IL-2, IL-4, granzyme B, perforin, IFN-γ, TNF-α) upon specific recognition of GD_2_-expressing target cells, but not after co-cultivation with a control cell line lacking the target. In addition, TRUCKs induced release of the engineered cytokine IL-18 in an antigen-dependent manner and mediated a very low background secretion upon co-cultivation with target-negative cells or spontaneous release without target cells. The risk of toxicity for IL-18 TRUCKs is different to IL-12 TRUCKs that revealed severe toxicity of T cells engineered with inducible IL-12 in a melanoma mouse model due to off-target cytokine secretion (4). However, other studies with IL-12 TRUCKs reported a safe administration into mice and such toxicities were not observed for IL-18 TRUCKs (2, 3, 5, 51). GMP-compliant manufactured IL-18 TRUCKs targeting GD_2_ showed a high cytotoxic capability and were able to eliminate tumor cells while forming clusters around target cells. IL-18 TRUCKs show higher toxicities against high GD_2_-expressing target cells confirming the *in vitro* studies by Wiebel et al. (52).

To accurately quantify and confirm cytotoxicity towards target cells, we combined different methodologies including the label-free, real-time monitoring by impedance measurements, flow cytometric analysis allowing for concurrent phenotypic evaluation of TRUCKs, and detection of pro-inflammatory cytokines as indirect and cytotoxic mediators as direct parameter of cell lysis in the co-culture. Kiesgen (53) *et al.* comprehensively compare the power and limitation of different cytotoxicity assays and emphasize that especially impedance-based assays display a superior sensitivity and signal-to-background ratio over the “gold standard” ^51^chromium-release assay making it possible to evaluate even low E:T ratios as most appropriate to resemble physiological conditions. Such low E:T ratios would be interesting to evaluate in further experiments, since the manufactured TRUCKs exhibited rapid elimination of SH-SY5Y cells in the lowest E:T ratio of 1:1. Moreover, *in vitro* tests using repeated antigen stimulation or inclusion of immunosuppressive factors present in the TME are increasingly being used and could give an insight about persistence and exhaustion level of the generated TRUCKs upon high antigen stress.

We show similar manufacturing of clinical-scale and laboratory-scaled IL-18 TRUCKs concerning transduction and amplification efficiency and cellular functionality. After cryopreservation of the T cell products, the specificity and cytotoxicity of TRUCKs was maintained. Attempts to treat solid tumors with redirected T cells have largely failed so far, with very few patients responding and with only transient and partial tumor regression (17, 18, 48, 54–57). The poor clinical outcome is thought to be due at least in part to an unfavorable environment in the tumor tissue that suppresses CAR T cell responses. TRUCK-secreted cytokine IL-18 led to increased recruitment of monocytes and NK cells in an *in vitro* cell migration assay. This may contribute to reprogramming the tumor stroma towards a more favorable environment for CAR T cell function, thereby enhancing their efficacy in the treatment of solid tumors. Furthermore, IL-18 was shown to polarize TRUCKs towards more potent pro-inflammatory effector cells that do not drive into functional exhaustion in the long term (5).

In conclusion, GMP-compliant manufacturing of IL-18 TRUCKs targeting GD_2_ using the automated closed CliniMACS Prodigy^®^ system is feasible and enables the manufacturing of a sufficient number of cells for clinical application. The automatic mode of operation improves standardization and robustness of the manufacturing process. This benefits the manufacturing at different sites for an academia-initiated multicenter trial. The smooth adaption of the process established and validated for the manufacturing of CAR T cells to generate IL-18 TRUCKs encourages the translation of the procedure to other cells and targets.

## Data Availability Statement

The original contributions presented in the study are included in the article/**Supplementary Material**. Further inquiries can be directed to the corresponding authors.

## Ethics Statement

For this study the donors gave their written informed consent and approval was granted by the corresponding ethics committee (No. 2829-2015). The study was approved by the ethics committee of Hannover Medical School (2519-2014, 2830-2015, 3639-2017).

## Author Contributions

Conceptualization: RE, WG, BE-V, AD, UK, KZ, and ASc. Methodology: AD, WG, RE, MM, AM-E, CK, ASt, KZ, and ASc. Formal analysis: WG, AD, RE, AM-E, MM, CK, ASt, KZ, and KA. Resources: HL, NS, and CR. Writing original draft application: WG, AD, RE, and AM-E. Writing review and editing: all authors. Supervision: HA, LA, TM, RB, LG, ASc, BE-V, and UK. Funding acquisition: BE-V, HA, TM, RB, ASc, UK, and CR. All authors have read and agreed to the published version of the manuscript.

## Funding

This work was supported by the following grants: “From CARs to TRUCKs: Induction of a concerted antitumor immune response by engineered T cells” (Deutsche Krebshilfe/German Cancer Aid-Priority Program in Translational Oncology #111975) and DFG-funded SFB738 (projects C4 & C9) and Cluster of Excellence REBIRTH (EXC62/2).

## Conflict of Interest

HA, ASc, and KZ have submitted a patent application describing the “all-in-one” TRUCK vector technology. The University Medicine of Greifswald licensed Ganglidiomab to AnYxis Immuno-oncology for commercialization.

The remaining authors declare that the research was conducted in the absence of any commercial or financial relationships that could be construed as a potential conflict of interest.

## Publisher’s Note

All claims expressed in this article are solely those of the authors and do not necessarily represent those of their affiliated organizations, or those of the publisher, the editors and the reviewers. Any product that may be evaluated in this article, or claim that may be made by its manufacturer, is not guaranteed or endorsed by the publisher.
