# Supplementary material for: GMP-Compliant Manufacturing of TRUCKs: CAR T Cells targeting GD2 and Releasing Inducible IL-18
Source: Front Immunol. 2022 Mar 24;13:839783. doi: 10.3389/fimmu.2022.839783 (PMC8988144; doi:10.3389/fimmu.2022.839783)
Supplement: Supplementary file 1 [file DataSheet_1.pdf]

SUPPLEMENTARY FIGURES AND TABLES

Figure S1

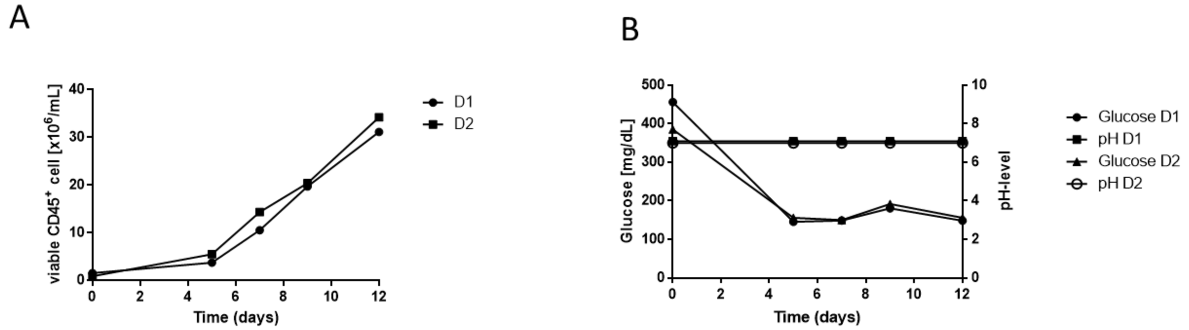

Supplementary Figure S1. Monitoring of viable CD45$^+$ cell concentration, pH-value and glucose. (A) Concentration of viable CD45$^+$ cells**.** The starting cell concentration was 1.5 x 10^6 D1 and 0.87 x 10 D2 viable CD45$^+$ cells/ml. During expansion and media exchange cell concentrations achieved 31.16 x 10^6 D1 and 34.2 x 10^6 D2 CD45$^+$ cells/ml. After increase of culture volume on day 1 (transduction) to 100 ml and on day 3 (culture wash) the volume kept constant at 200 ml. From day 3 after culture wash for removal of vector and activation reagent the culture was shaken automatically. **(B)** Monitoring of culture conditions with measurement of pH and concentration of glucose during 12-day cultivation.

Figure S2

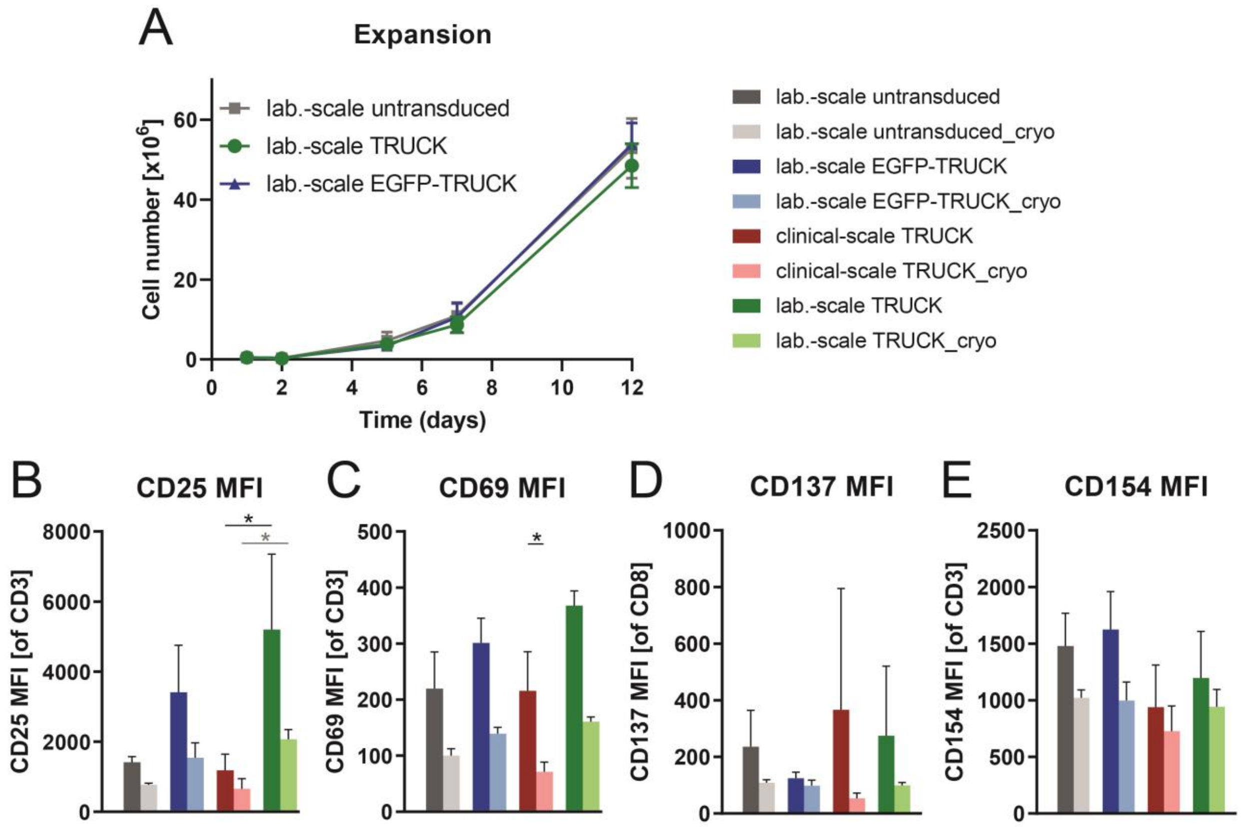

Supplementary Figure S2. Expansion of laboratory-scale TRUCKs and pre-activation of clinical- and laboratory-scale TRUCKs: IL-18 TRUCKs targeting GD_2 were generated using the CliniMACS Prodigy® (clinical-scale TRUCK; n=2) or under laboratory conditions (lab.-scale TRUCK; n=3). Untransduced T

cells (lab.-scale untransduced; n=3) as well as GD_2 TRUCKs with inducible EGFP expression (EGFP-TRUCK; n=3) served as control. The manufactured cells were characterized either directly after generation or after cryopreservation and thawing (cryo). **(A)** The expansion of untransduced TC and TRUCKs generated in the laboratory scale with a starting population of 6 x 10^5 cells was determined by cell counting. Data is shown as mean ± SD. **(B-E)** Expression of the activation markers **(B)** CD25 on $CD3^+$, **(C)** CD69 on $CD3^+$, **(D)** CD137 on $CD8^+$ and **(E)** CD154 on $CD4^+$ T cells as median fluorescence intensity (MFI). **(B-E)** Data is shown as mean ± SD. Statistical differences of large-scale TRUCKs directly after generation or cryopreservation as well as in comparison to all laboratory-scale manufactured cells were assessed by Kruskal-Wallis and Dunn's test, whereby only significant differences are shown (*$p \leq 0.05$).

Figure S3

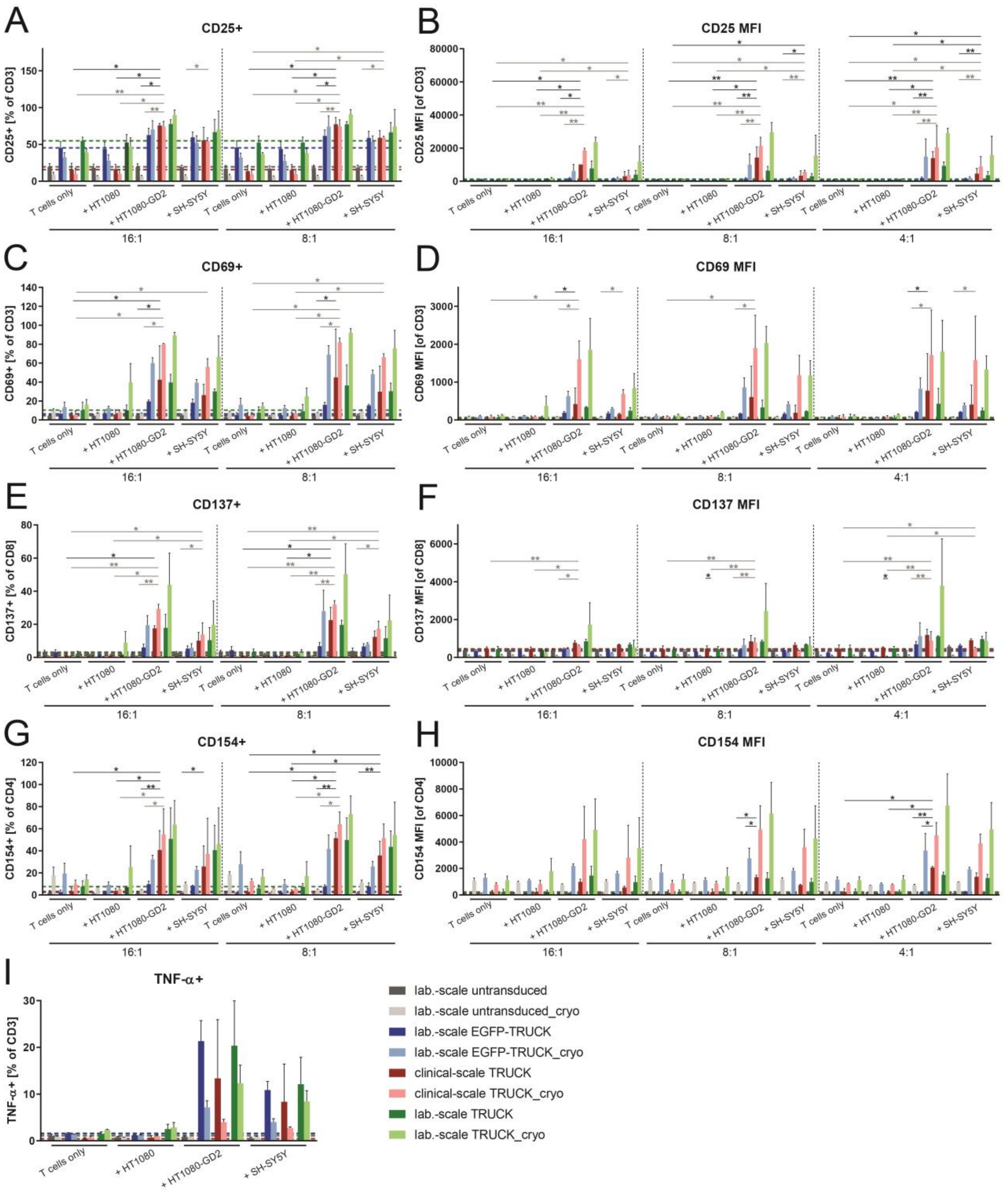

Supplementary Figure S3. Clinical-scale-manufactured IL-18 TRUCKs targeting GD$_2$ specifically respond to GD$_2^+$ target cells with an increase of activation marker expression and release of TNF-α: IL-18 TRUCKs targeting GD$_2$ were generated using the CliniMACS Prodigy® (clinical-scale TRUCK; n=2) or under laboratory conditions (lab.-scale TRUCK; n=3). Untransduced T cells (lab.-scale untransduced; n=3) as well as GD$_2$ TRUCKs with inducible EGFP expression (EGFP-TRUCK; n=3) served as control.

The manufactured cells were tested for GD_2-CAR-mediated activation either directly after the generation process (d12) or after cryopreservation and thawing (cryo) by co-cultivation with the indicated target cells for 48h in the indicated effector-to-target (E:T) ratios or cultivation alone (T cells only). **(A-E)** Expression of the activation markers **(A, B)** $CD25^+$ of $CD3^+$, **(C, D)** $CD69^+$ of $CD3^+$, **(E, F)** $CD137^+$ of $CD8^+$ and **(G, H)** $CD154^+$ of $CD4^+$ T cells as **(A, C, E, G)** frequency or **(B, D, F, H)** median fluorescence intensity (MFI). **(I)** Concentration of released TNF-α in the cell culture supernatants after 48h was assessed by LEGENDPlex™. **(A-I)** A dashed line indicates background levels of the respective expression by untransduced T cells (grey), EGFP-TRUCKs (blue), as well as clinical-scale (red) and laboratory-scale (green) TRUCKs cultured alone. Data is shown as mean ± SD. Statistical differences of large-scale TRUCKs co-cultured with different target cells directly after generation or cryopreservation as well as in comparison to all laboratory-scale manufactured cells were assessed by Kruskal-Wallis and Dunn's test, whereby only significant differences are shown ($*p \leq 0.05$, $**p \leq 0.01$).

Figure S4

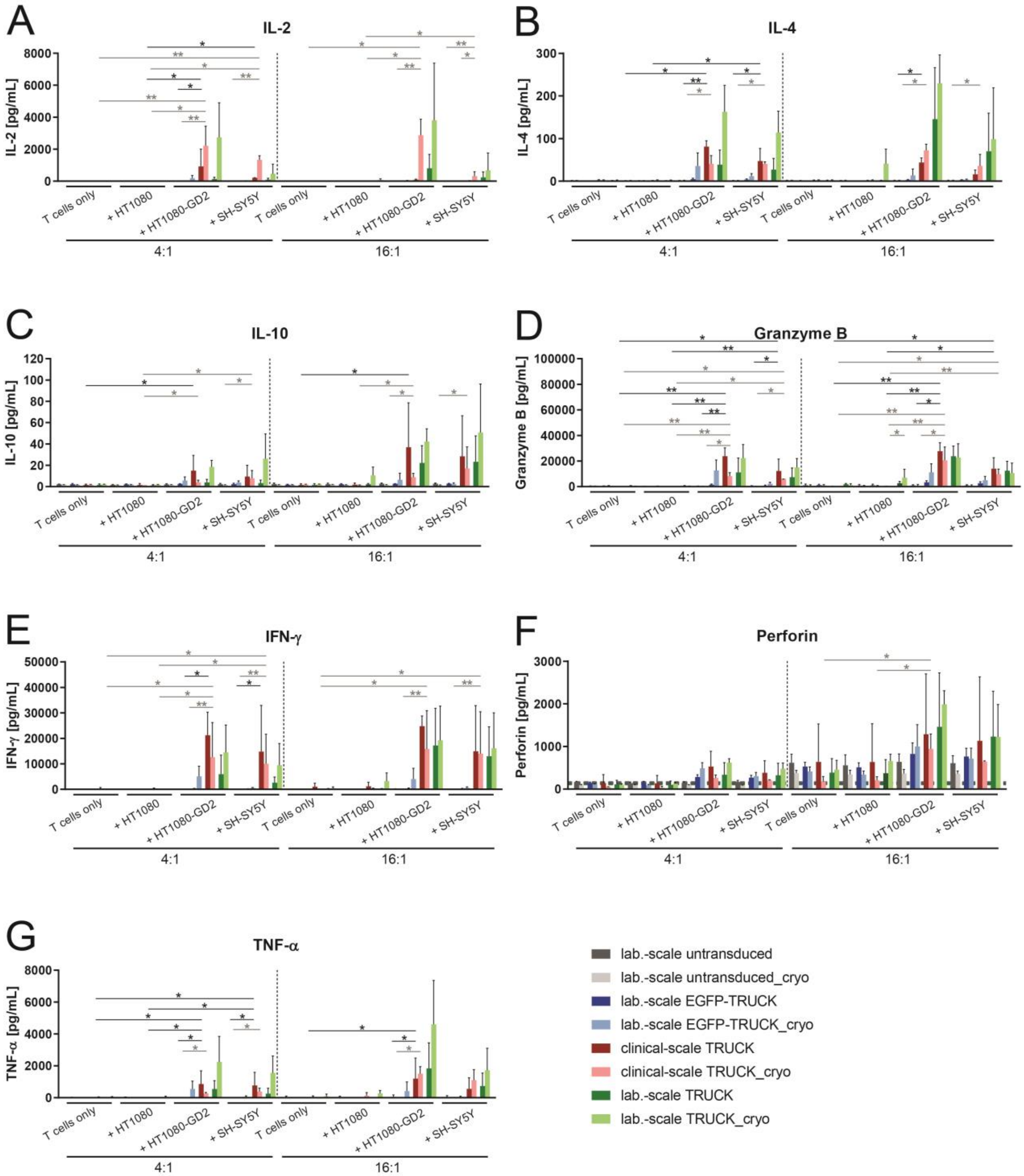

Supplementary Figure S4. Clinical-scale-manufactured IL-18 TRUCKs targeting GD_2 specifically react to target recognition with increased release of soluble mediators. IL-18 TRUCKs targeting GD_2 were generated using the CliniMACS Prodigy® (clinical-scale TRUCK; n=2) or under laboratory conditions (lab-scale TRUCK; n=3). Untransduced T cells (lab.-scale untransduced; n=3) as well as GD_2 TRUCKs with inducible EGFP expression (EGFP-TRUCK; n=3) served as control. The manufactured cells were tested for functionality either directly after the generation process (d12) or after cryopreservation and thawing (cryo) by co-cultivation with the indicated target cells in the indicated effector-to-target (E:T) ratios

or cultivation alone (T cells only). The concentration of released cytokines **(A)** IL-2, **(B)** IL-4, **(C)** IL-10, **(D)** granzyme B, **(E)** IFN-γ, **(F)** perforin, and **(G)** TNF-α in the cell culture supernatants after 48h was assessed by LEGENDPlex™. **(F)** A dashed line indicates background levels of the respective cytokine release by untransduced T cells (grey), EGFP-TRUCKs (blue), as well as clinical-scale (red) and laboratory-scale (green) TRUCKs cultured alone. **(A-G)** Data is shown as mean ± SD. Statistical differences of large-scale TRUCKs co-cultured with different target cells directly after generation or cryopreservation as well as in comparison to all laboratory-scale manufactured cells were assessed by Kruskal-Wallis and Dunn's test, whereby only significant differences are shown (*$p \leq 0.05$, **$p \leq 0.01$).

Figure S5

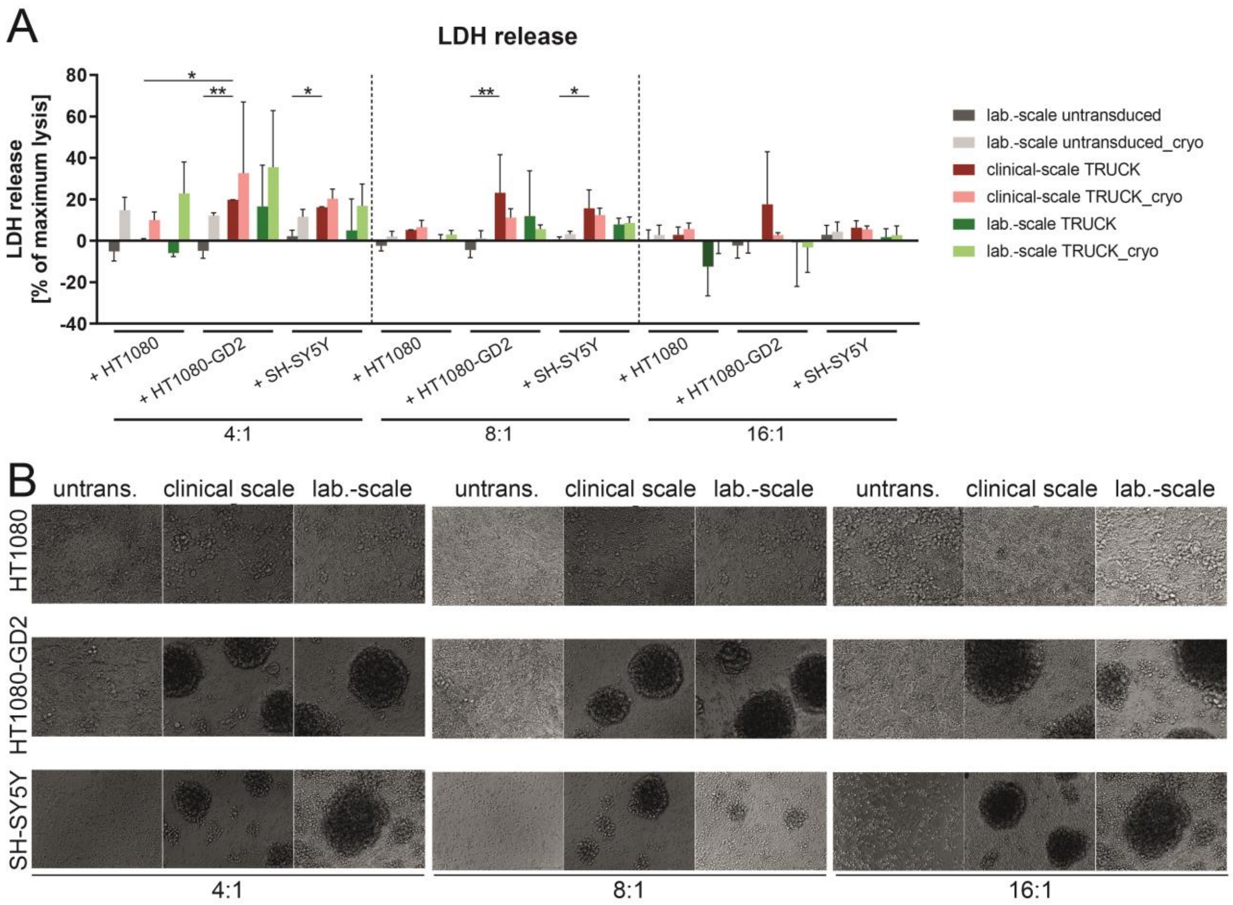

Supplementary Figure S5. Clinical-scale-manufactured GD_2-specific IL-18 TRUCKs specifically eliminate GD_2^+ target cells: IL-18 TRUCKs targeting GD_2 were generated using the CliniMACS Prodigy® (clinical-scale TRUCK; n=2) or under laboratory conditions (lab.-scale TRUCK; n=3). Untransduced T cells (lab.-scale untransduced; n=3) served as control. The manufactured cells were tested for cytotoxicity either directly after the generation process (d12) or after cryopreservation and thawing (cryo) by co-cultivation with the indicated target cells and in the indicated effector-to-target (E:T) ratios for 48h. **(A)** Release of lactate dehydrogenase (LDH) into the cell culture supernatant as an indicator of the killing of target cells by T cells. LDH levels are expressed as a percentage of the maximum lysis level obtained using controls lysed with 1% Triton X-100. Data is shown as mean ± SD. Statistical differences of large-scale TRUCKs co-cultured with different target cells directly after generation or cryopreservation as well as in comparison to all laboratory-scale manufactured cells were assessed by Kruskal-Wallis and Dunn's test, whereby only significant differences are shown (*$p \leq 0.05$, **$p \leq 0.01$). **(B)** Representative transmitted-

light microscope images of co-cultures of cryopreserved effector cells with target cells taken by an Olympus IX81 microscope combined with a digital B/W camera using 10x objective lenses.

<u>Figure S6</u>

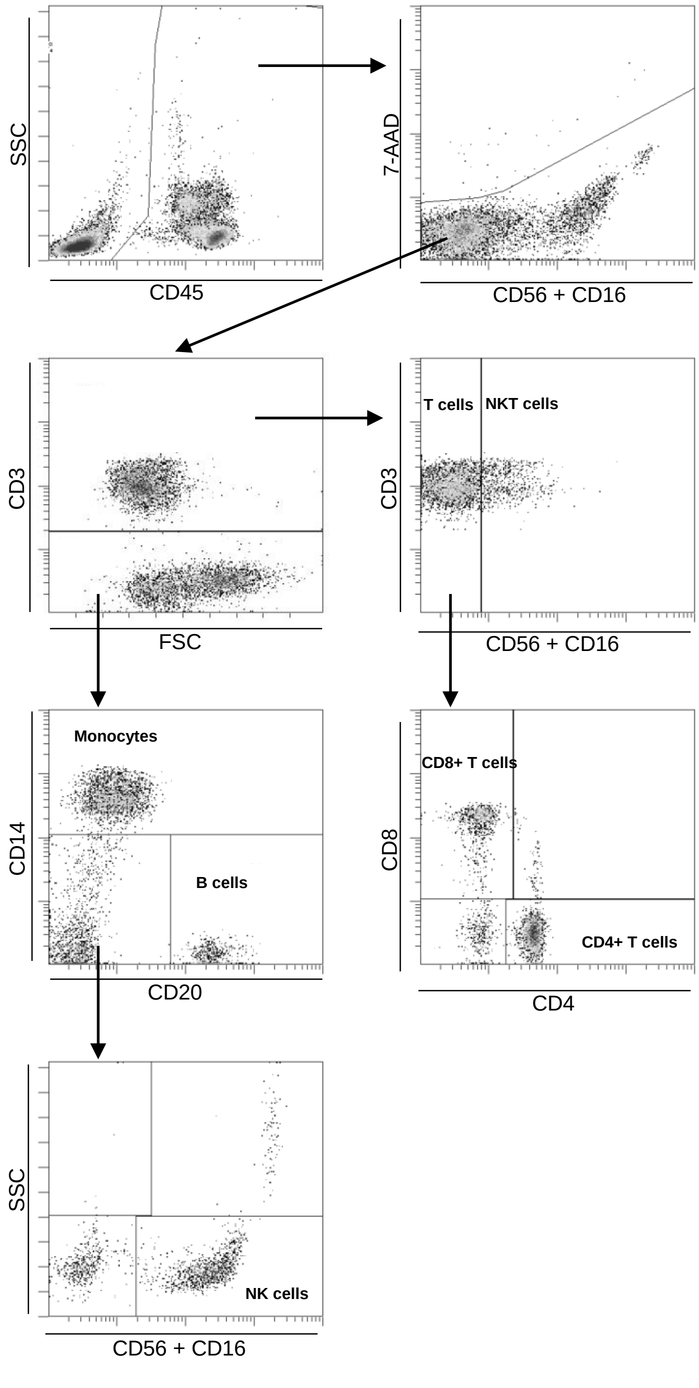

Supplementary Figure S6. Gating strategy to determine cellular composition during clinical-scale process manufactured IL-18 TRUCKs by flow cytometry exemplarily shown for lymphapheresis on process day -1: Cells were stained for surface antigens and analyzed based on their expression of different markers: T cells (7-AAD$^-$ CD45$^+$ CD3$^+$ CD56$^-$/CD16$^-$), CD4$^+$ T cells (7-AAD$^-$ CD45$^+$ CD3$^+$ CD56$^-$/CD16$^-$ CD4$^+$), CD8$^+$ T cells (7-AAD$^-$ CD45$^+$ CD3$^+$ CD56$^-$/CD16$^-$ CD8$^+$), NKT cells (7-AAD$^-$ CD45$^+$ CD3$^+$ CD56$^+$/CD16$^+$), NK cells (7-AAD$^-$ CD45$^+$ CD3$^-$ CD56$^+$/CD16$^+$), B cells (7-AAD$^-$ CD45$^+$ CD3$^-$ CD20$^+$), monocytes (7-AAD$^-$ CD45$^+$ CD3$^-$ CD14$^+$).

Figure S7

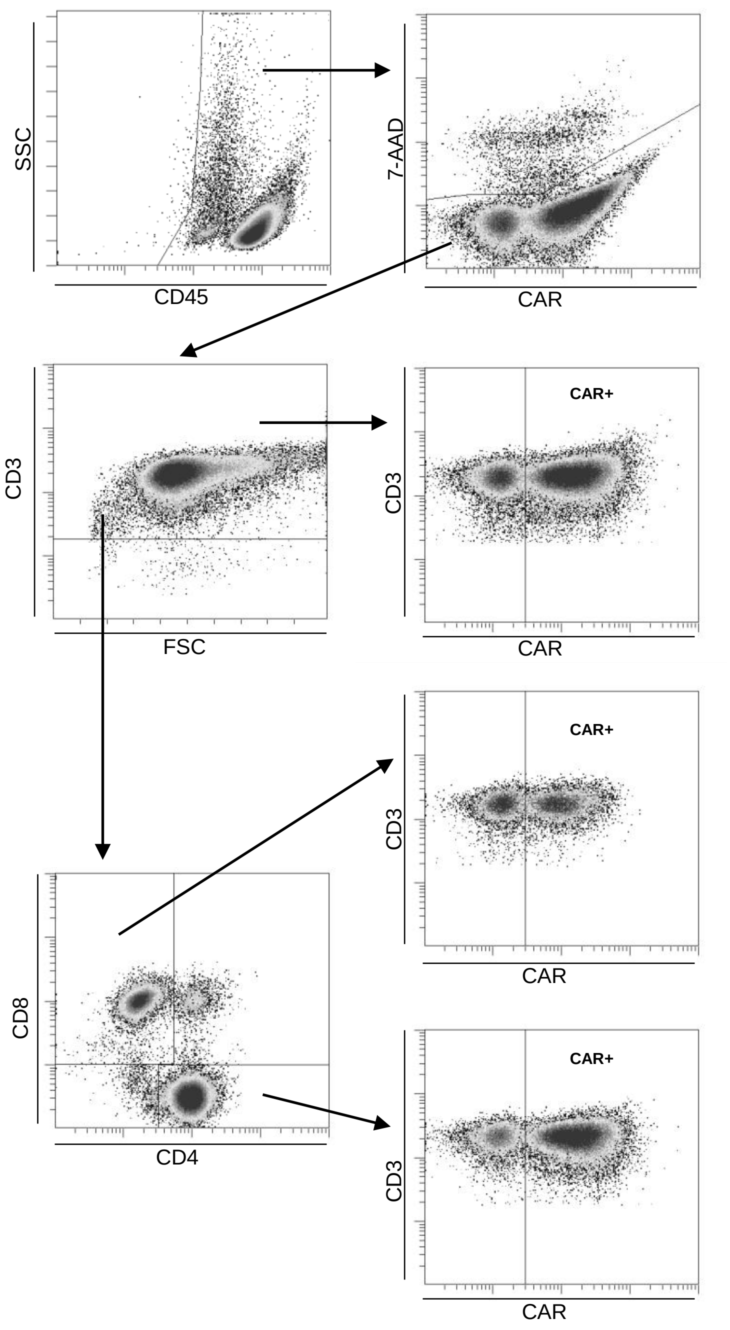

Supplementary Figure S7. Gating strategy to determine transduction rate during clinical-scale process manufactured IL-18 TRUCKs by flow cytometry exemplarily shown for the final product on process day 12: Cells were stained for surface antigens and analyzed based on their expression of different markers: $CD3^{+}CAR^{+}$ (7-AAD^{-} $CD45^{+}$ $CD3^{+}$ CAR^{+}), $CD4^{+}CAR^{+}$ (7-AAD^{-} $CD45^{+}$ CD3+ $CD4^{+}$ CAR^{+}), $CD8^{+}CAR^{+}$ (7-AAD^{-} $CD45^{+}$ $CD3^{+}$ $CD8^{+}$ CAR^{+}).

Figure S8

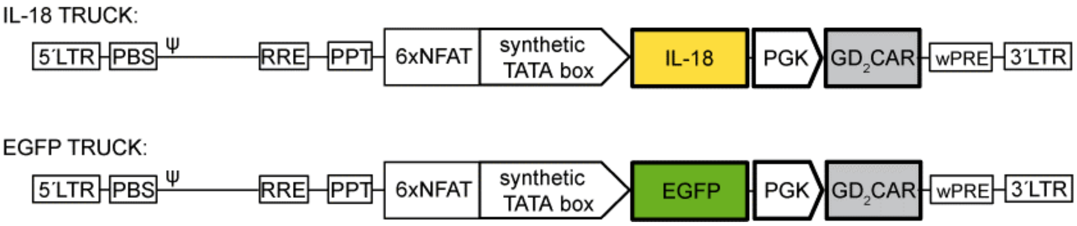

Supplementary Figure S8. Schematic map of the lentiviral self-inactivating (SIN) IL-18 and EGFP TRUCK vector constructs: The constitutive GD_2 CAR expression is driven by a phosphoglycerate kinase (PGK) promoter and the inducible IL18 expression, or EGFP expression respectively, is driven by an NFAT promoter element consisting of six consensus NFAT repeats fused to a synthetic TATA box. Indicated are the primer binding site (PBS), packaging signal (ψ), rev-responsive element (RRE), central poly-purine tract (cPPT), and woodchuck hepatitis virus post-transcriptional regulatory element (wPRE) (26).

Supplementary Table S1. List of antibodies (w/o Ganglidiomab-PE Ab) used for **(A)** clinical-scale cell composition, **(B)** clinical-scale T cell phenotype and **(C)** laboratory-scale analyses.

A

Antibody	Clone	Company
**CD3-FITC**	REA613	Miltenyi Biotec
**CD4-VioBlue**	VIT4	Miltenyi Biotec
**CD8-APC-Vio770**	BW135/80	Miltenyi Biotec
**CD14-APC**	TÜK4	Miltenyi Biotec
**CD16-PE**	REA423	Miltenyi Biotec
**CD20-PE-Vio770**	REA780	Miltenyi Biotec
**CD45-VioGreen**	REA747	Miltenyi Biotec
**CD56-PE**	REA196	Miltenyi Biotec

B

Antibody	Clone	Company
**CD3-PacificBlue**	UCHT1	Beckman Coulter
**CD4-Krome Orange**	13B8.2	Beckman Coulter
**CD8-APC-Vio770**	BW135/80	Miltenyi Biotec
**CD45RO-FITC**	UCHL1	Becton Dickinson
**CD95-APC**	DX2	Becton Dickinson
**CCR7-PE-Vio615**	REA546	Miltenyi Biotec

C

Antibody	Clone	Company
**CD3-PerCP**	SK7	BioLegend
**CD4-BV510**	RPA-T4	BioLegend
**CD4-PE-Cy7**	SK3	BioLegend
**CD8-AF700**	SK1	BioLegend
**CD8-APC**	SK1	BioLegend
**CD25-BV421**	BC96	BioLegend
**CD69-BV605**	FN50	BioLegend
**CD137-PE-Cy7**	4B4-1	BioLegend
**CD154-APC-Cy7**	24-31	BioLegend
**TNF-α-APC**	Mab 11	BioLegend
